# A chromosome-level genome assembly for *Onobrychis viciifolia* reveals gene copy number gain underlying enhanced proanthocyanidin biosynthesis

**DOI:** 10.1038/s42003-023-05754-6

**Published:** 2024-01-05

**Authors:** Junyi He, Danyang Tian, Xue Li, Xuemeng Wang, Tingting Wang, Ziyao Wang, Hui Zang, Xiaofan He, Tiejun Zhang, Quanzheng Yun, Rengang Zhang, Jishan Jiang, Shangang Jia, Yunwei Zhang

**Affiliations:** 1 College of Grassland Science and Technology, China Agricultural University, 100193 Beijing, China; 2 School of Grassland Science, Beijing Forestry University, 100083 Beijing, China; 3 Department of Bioinformatics, Ori (Shandong) Gene Science and Technology Co., Ltd., Weifang, 261322 China

**Keywords:** Secondary metabolism, Plant evolution

## Abstract

Sainfoin (*Onobrychis viciifolia*), which belongs to subfamily Papilionoideae of Leguminosae, is a vital perennial forage known as “holy hay” due to its high contents of crude proteins and proanthocyanidins (PAs, also called condensed tannins) that have various pharmacological properties in animal feed, such as alleviating rumen tympanic disease in ruminants. In this study, we select an autotetraploid common sainfoin (2n = 4x = 28) and report its high-quality chromosome-level genome assembly with 28 pseudochromosomes and four haplotypes (~1950.14 Mb, contig N50 = 10.91 Mb). The copy numbers of genes involved in PA biosynthesis in sainfoin are significantly greater than those in four selected Fabales species, namely, autotetraploid *Medicago sativa* and three other diploid species, *Lotus japonicus*, *Medicago truncatula*, and *Glycine max*. Furthermore, gene expansion is confirmed to be the key contributor to the increased expression of these genes and subsequent PA enhancement in sainfoin. Transcriptomic analyses reveal that the expression of genes involved in the PA biosynthesis pathway is significantly increased in the lines with high PA content compared to the lines with medium and low PA content. The sainfoin genome assembly will improve our understanding of leguminous genome evolution and biosynthesis of secondary metabolites in sainfoin.

## Abstract (web summary)

A chromosome-level genome assembly for *Onobrychis viciifolia* (autotetraploid sainfoin) combined with transcriptomic data provides insights into how proanthocyanidins are synthesized and Onobrychis evolution.

## Introduction

Sainfoin (*Onobrychis viciifolia*) is a significant perennial forage that belongs to subfamily Papilionoideae of Leguminosae and is known as “holy hay” due to its >15% crude protein content^1^. It is mostly tetraploid (2n = 4x = 28) and cross-pollinated, which leads to high levels of genetic diversity and phenotypic variations^2–5^. For example, there were significant differences in winter survival rate, dry matter yield, seed yield, and stem number among sainfoin germplasms^6,7^. In addition, higher levels of crude protein, moderate levels of soluble sugar, and lower levels of acid and neutral detergent fiber contributed to the palatability of livestock feed made from *O. viciifolia*. It was reported that the palatability of *O. viciifolia* to ruminants and the animal productivity level per unit feed consumption are equivalent to or higher than those of alfalfa (*Medicago sativa*)^8^. Meanwhile, proanthocyanidins (PAs), also called condensed tannins (CTs), are highly abundant in *O. viciifolia* and distributed in almost all organs except for roots and cotyledons^9–11^. Studies have shown that PAs play multiple key roles in ruminant feeding^12^, including alleviating rumen tympanic disease in ruminants, improving protein utilization, and increasing antiparasitic activity in the rumen^12–18^. Owing to the high PA content and its benefits, *O. viciifolia* has the potential to be used for forage feeding and industrial production of PAs.

PAs can be found in a variety of forage species of Fabaceae, including big trefoil and bird’s-foot trefoil in *Lotus*^15^, white clover and red clover in *Trifolium*^19,20^, alfalfa in *Medicago*^21,22^, and sainfoin^23^. In previous studies, researchers discovered that the PA content of *O. viciifolia* was ~80 g/kg, while that of *M. sativa* was ~0.5 g/kg^15^. Meanwhile, PA was found in all parts of *O. viciifolia*, while in *M. sativa*, it was enriched mainly in the seed coat^15^. Despite the variations in PA contents, these species might share similar PA biosynthetic pathways, according to studies in *Arabidopsis thaliana*, *Medicago truncatula* and other model plants^24,25^. PAs are produced through phenylpropanoid and flavonoid pathways and share most of the biosynthetic pathways with anthocyanins, only with a split downstream^26^. Meanwhile, recent studies have shown that a large number of transcription factors (TFs), including R2-R3 MYB, bHLH, and WD40 TFs, are involved in facilitating multiple enzymatic steps in PA biosynthesis^27,28^. However, the orthologous genes for PA biosynthesis in the *O. viciifolia* genome are unknown.

It is widely believed that sainfoin originated in Asia, was introduced to Europe via Arabs in the fourteenth century, and was used for hay and seed production on a large scale in the twentieth century^29^. *O. viciifolia* phylogenetically falls into the clade of common leguminous species (Hologalegina, Papilionoideae, Fabaceae), including *Pisum sativum*, *M. sativa*, and *Cicer arietinum*. To date, multitudinous genome assemblies of leguminous species, including chickpea (*C. arietinum*), cultivated soybean (*Glycine max*), alfalfa (*M. sativa*) and Chinese milk vetch (*Astragalus sinicus*)^30–33^, have greatly promoted our understanding of the evolutionary history of Fabaceae. Alfalfa has four genome assemblies available, including those for “Xinjiang Daye”, *M. sativa* ssp. *caerulea*, “Zhongmu No. 1”, and “Zhongmu No. 4”. The latest genome version of Zhongmu No. 4 included four haplotypes for 32 chromosomes (2n = 4x = 32), with a genome size of 2.74 Gb and contig N50 of 2.06 Mb, showing multiple genome evolution events in alfalfa compared to *M. truncatula*^32,34–36^. Chromosome-level assembly of autopolyploid genomes is a major challenge, especially for species with a large genome size. Similar to alfalfa, *O. viciifolia* is also an autotetraploid legume forage, but its genome is not available for exploring genome evolutionary history. Available genomic resources for *O. viciifolia* are rare, and scientists have revealed the genetic diversity and phylogenetic relationships of sainfoin by using amplified fragment length polymorphisms (AFLPs), inter-simple sequence repeats (ISSRs), simple sequence repeats (SSRs), expressed sequence tag-derived simple sequence repeats (EST-SSRs), and single nucleotide polymorphisms (SNPs)^37–41^. The lack of a high-quality, chromosome-level genome assembly for sainfoin has hampered its genetic research and improvement, although the complete chloroplast genome sequence of sainfoin and some transcriptomic resources are available^42^.

**Fig. 1:**
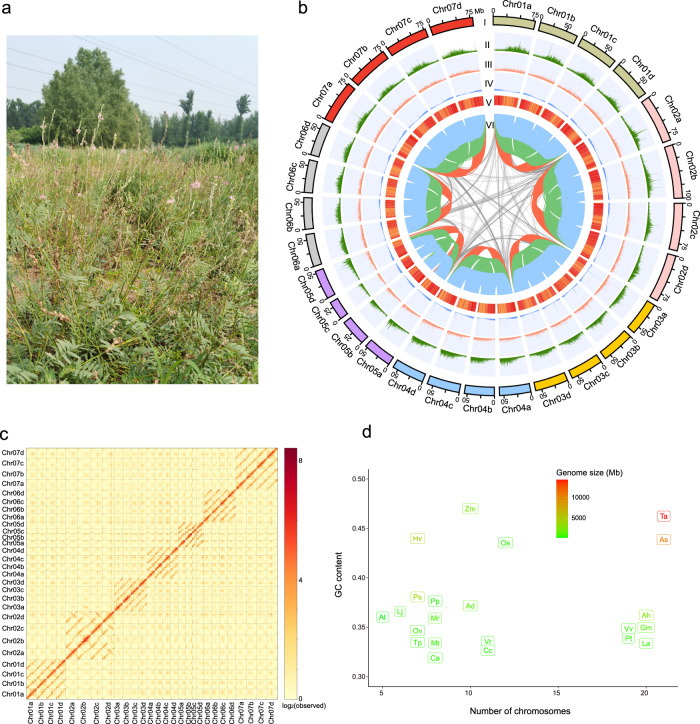
Distribution of genomic features within the genome assembly of *O. viciifolia*. **a** *O. viciifolia* grown in the field, **b** genomic features of *O. viciifolia*. **I,** Twenty-eight chromosomes of *O. viciifolia*; **II,** Density of Gypsy elements; **III,** Density of Copia elements; **IV,** Density of genes; **V,** GC content; **VI,** Links of collinear gene blocks of *O. viciifolia* among four haplotypes. Blue ribbons indicate synteny blocks between chra and chrb, chrb and chrc, and chrc and chrd; green ribbons indicate synteny blocks between chra and chrc and chrb and chrd; and red ribbons indicate synteny blocks between chra and chrd. Each sliding window was 100 kb. **c** Hi-C interaction heatmap of 28 chromosomes in *O. viciifolia*. **d** GC content vs. chromosome number of Fabaceae and non-Fabaceae species, At-*A. thaliana*, Pp-*P. persica*, Pt-*P. trichocarpa*, Vv-*V. vinifera*, Ad-*A. duranensis*, Ah-*A. hypogaea*, La-*L. angustifolius*, Cc-*C. cajan*, Vr-*V. radiata*, Gm-*G. max*, Lj-*L. japonicus*, Ov-*O. viciifolia*, Ca-*C. arietinum*, Ps-*P. sativum*, Tp-*T. pratense*, Mr-*M. ruthenica*, Mt-*M. truncatula*, Zm-*Z. mays*, Os-*O. sativa*, Hv-*H. vulgare*, Ta-*T. aestivum*, As-*A. sativa*.

In this study, we present a high-quality, chromosome-level genome assembly of common sainfoin (Fig. 1a). The assembly contains 28 pseudochromosomes with four haplotypes. We confirmed that gene expansion, which was driven by a combination of autotetraploidization, whole-genome duplication, and fragmental duplication events, increased the expression of genes associated with the production of PAs, which contributed to the enhanced PA content in *O. viciifolia* leaves. Our results provide a basis for understanding the genomic evolution of the genus *Onobrychis* and legume species and for accelerating genetic breeding in *Onobrychis* species and functional genomic studies in *O. viciifolia*.

## Results

### Assembly and annotation of the sainfoin genome

It was noted previously that a single haplotype of the autotetraploid genome lost many genes^43^. Therefore, we sought to assemble the four haplotypes of autotetraploid *O. viciifolia*. We sequenced the genomic DNA of one *O. viciifolia* plant by combining Oxford Nanopore technology (ONT), Illumina NovaSeq and Hi-C and generated ~200 Gb (coverage: ~109.89 X), 175 Gb (coverage: ~96.15 X), and 300 Gb (coverage: ~164.84 X) of data, respectively (Supplementary Table 1). Using *K*-mer analysis (*k* = 17), the genome size of sainfoin was estimated to be ~1821.52 Mb, which is close to the result of ~1851.75 Mb obtained by flow cytometry (Supplementary Fig. 1a, b). To accurately assemble the sainfoin genome, we first performed the preliminary assembly of four haplotypes based on ONT clean reads and used short reads to polish the assembled contigs. First, we preliminarily assembled the contigs based on the Hi-C data and 3D-DNA, and the process effectively corrected some errors; however, there were also many errors in the output result. Then, we corrected the errors manually by Juicebox, and the process did not have a quantitative standard but followed expectations based on the principle that an interaction signal with a shorter distance is stronger than one with a longer distance in the Hi-C auxiliary assembly process. Based on our optimized pipeline, the nuclear assembly (~1950.14 Mb) and phasing placed 1044 contigs (N50 = 10.92 Mb) onto 28 pseudochromosomes (Supplementary Table 2), which represented four haplotypes with seven chromosomes (4 × 7 chromosomes, ~1892.58 Mb) and unplaced contigs (57.56 Mb) (Fig. 1b, c, Supplementary Fig. 2 and Supplementary Table 3). However, we found a low level of switch error and collapsed contig (Supplementary Fig. 2) due to the homologous regions among the four haplotypes when the assembled contigs were placed in 28 pseudochromosomes. Based on the ONT long reads, we found 770 collapsed regions and 40 switch errors by using Inspector program, whose lengths reached 0.17 Mb and 0.018 Mb respectively (Supplementary Fig. 3). The GC content of the entire genome was 34.62% (Supplementary Table 4), and the average content of the four haplotypes was 34.61%, which was close to the 34.97% of *Glycine max* in Fabaceae and 34.86% of *Vitis vinifera* in Pentapetalae (Fig. 1d, Supplementary Table 5 and Supplementary Data 1). Despite collapsed contigs and switch errors, the four haplotypes (labeled Haplotypes A ~ D) showed similar total lengths ranging from 458.4 Mb to 482.9 Mb (Fig. 1c and Supplementary Table 5).

We identified 3,147,173 repetitive sequences with a total length of 1239.64 Mb (63.55% of the whole genome assembly) (Supplementary Table 6). We also annotated 1,033,972 long terminal repeat (LTR) retrotransposons (~36.14%), which were the most abundant transposable elements (TEs) in the genome. There were 474,059 simple repeat sequences, 381,092 Gypsy elements, and 218,580 Copia retrotransposons in the 28 pseudochromosomes. Gypsy and Copia LTR retrotransposons accounted for 17.4% and 7.7%, respectively (Supplementary Table 6). Combining ab initio prediction and evidence-based methods, 109,998 high-confidence genes were identified, which contained 719,988 exon domains (Supplementary Table 7). The average number of genes of the four haplotypes was 27,284, similar to the 27,571 of *Cicer arietinum* and 28,251 of *Lotus japonicus* (Supplementary Table 5)^30,44^.

To evaluate genome assembly quality, we downloaded RNA-Seq (Supplementary Table 8) data from the NCBI SRA and mapped the clean reads to the genome, along with short and long DNA reads. The mapping rates were 99.0%, 97.1%, and 91.7% for short DNA reads, long DNA reads, and RNA-Seq short reads, respectively (Supplementary Table 9). We further identified 1315 (91.3%) of the 1440 total core genes in the BUSCO (Benchmarking Universal Single-Copy Orthologs) analysis based on embryophyta_odb9, including 108 single-copy BUSCOs (7.5%), 1207 duplicated BUSCOs (83.8%), 23 fragmented BUSCOs (1.6%), and 102 missing BUSCOs (7.1%), while the integrated proteins covered 97.2% of the complete core genes (Supplementary Table 10). We also evaluated four haplotypes of the genome assembly by BUSCOs, and the results showed that 91.3% ~ 93.0% of BUSCOs were matched for the annotated proteins of four haplotypes (Supplementary Table 5). We annotated 99,518 coding genes against the databases (90.47% of the total genes), and the majority of genes received a functional assignment, for example, 98.51% for the nonredundant Nr protein sequence database, 97.86% for Clusters of Orthologous Genes (COG), and 98.67% for the protein database translated from EMBL (TrEMBL) (Supplementary Table 11). These results indicated high integrity and quality of the genome assembly. Meanwhile, by assembling the four haplotypes, we obtained 99,145 annotated genes that could be anchored to the 28 chromosomes and accounted for 90.84% of the total number of genes anchored to the chromosomes (Supplementary Fig. 4 and Supplementary Tables 12–15). We also assessed the assembly based on the long terminal repeat assembly index (LAI). The LAIs were 6.49, 5.79, 7.05, and 4.78 for the four haplotypes (Supplementary Table 16). This result suggested that the four haplotypic assemblies reached the medium level, while the LAI was 3.67 for *C. arietinum*, 3.79 for *C. cajan*, and 10.72 for *M. truncatula*. And the average quality value of the whole genome reached up to 33.4, with an error rate of 0.00046 and the completeness of 93.98% (Supplementary Table 17).

By considering the genome information of the four haplotypes of autotetraploid *O. viciifolia*, we obtained a relatively complete genome for *O. viciifolia* (Fig. 1b and Supplementary Tables 12 and 13). Although there were some errors in phasing the highly similar contigs of the four haplotypes (Supplementary Figs. 2 and 3), the assembled genome of the four haplotypes of *O. viciifolia* is expected to have a positive impact on genetic and molecular biology research.

### Gene family and evolutionary analysis

**Fig. 2:**
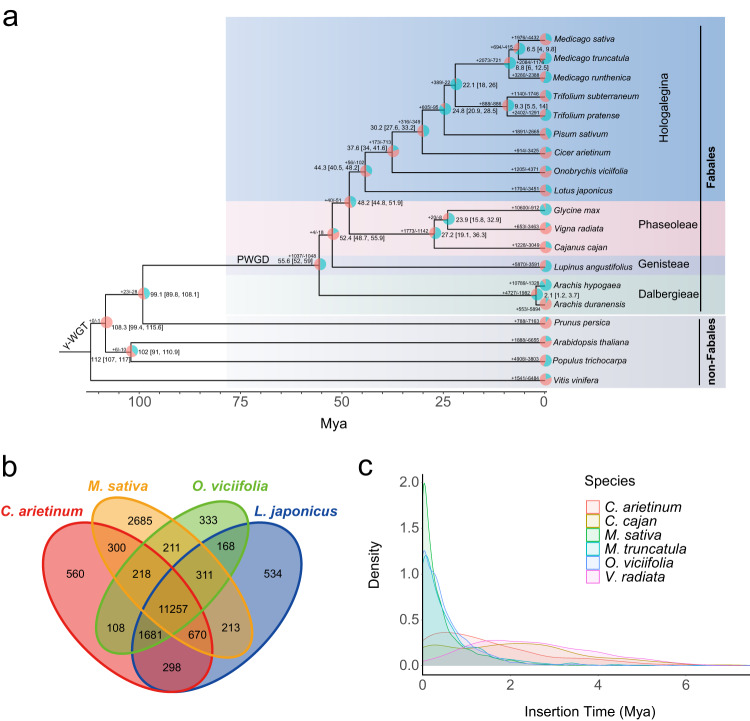
Gene family and phylogenetic tree of *O. viciifolia* and selected species. **a** Phylogenetic tree of *O. viciifolia* and 18 other Fabales and non-Fabales species. The numbers at each node represent the divergence time, and the time range in brackets is based on the 95% confidence interval. The pie chart represents the expansion and contraction of gene families, with blue for expansion and red for contraction, and the numbers next to the pie chart represent the number of gene families with expansion and contraction. **b** Venn diagram for gene families in *C. arietinum*, *M. sativa*, *O. viciifolia*, and *L. japonicus*. **c** LTR insertion time and density in six Fabales plants.

Based on 448 single-copy genes and their protein sequences obtained from OrthoFinder2, we built a phylogenetic tree of 19 representative species primarily from Fabales and showed that *O. viciifolia* phylogenetically neighbors *L. japonicus* and *C. arietinum* (Fig. 2a). We estimated the divergence time for this phylogenetic tree and found that in the Hologalegina clade, the divergence time of *O. viciifolia* from *L. japonicus* was ~44.3 million years ago (Mya), and *O. viciifolia* speciation started ~37.6 Mya, which was much earlier than that of the other Hologalegina species in the tree, for example, ~6.5 Mya for the split of *M. sativa* (Zhongmu No. 1) and *M. truncatula*. In contrast, the Fabales clade was estimated to have diverged from non-Fabales species ~99.1 Mya. We discovered 27,749 homologous gene families by comparing the common sainfoin (*O. viciifolia*) genome with the genomes of other species in the phylogenetic tree and 1205 expanded and 4371 contracted gene families in *O. viciifolia*, which were similar to those of other Fabales species. KEGG enrichment analysis revealed that the expanded gene families were mainly enriched in the pathways of biosynthesis of cofactors, amino sugar and nucleotide sugar metabolism, and flavonoid biosynthesis, while the contracted gene families were enriched in the pathways of plant hormone signal transduction, biosynthesis of various plant secondary metabolites, and cyanoamino acid metabolism (Supplementary Fig. 5). Furthermore, we identified the lowest number of multiple-copy gene families in *O. viciifolia* compared to *L. japonicus*, *C. arietinum*, and *M. sativa* (Supplementary Fig. 6), which was contrary to the higher number of copies of genes involved in PA biosynthesis (see the following results). Meanwhile, we compared gene families among the four selected species and found 11,257 common conserved gene clusters and 333 unique gene families in *O. viciifolia* (Fig. 2b). In the KEGG enrichment analysis, the unique gene families in *O. viciifolia* were enriched in the pathways of “zeatin biosynthesis”, “taurine and hypotaurine metabolism”, and “alanine, aspartate and glutamate metabolism” (Supplementary Table 18).

### LTR insertion and genome evolution

We discovered numerous long terminal repeats (LTRs) in the *O. viciifolia* genome, and we calculated the LTR insertion time for six Fabales species: *O. viciifolia*, *M. sativa*, *M. truncatula*, *C. arietinum*, *C. cajan*, and *V. radiata*. The results showed that the peak insertion time of *O. viciifolia* was ~0.033 million years ago (Mya), which was similar to that of *M. sativa* (~0.038 Mya) and *M. truncatula* (~0.077 Mya) and more recent than those of *C. arietinum* (~0.529 Mya), *C. cajan* (~2.188 Mya) and *V. radiata* (~1.653 Mya) (Fig. 2c and Supplementary Data 2).

**Fig. 3:**
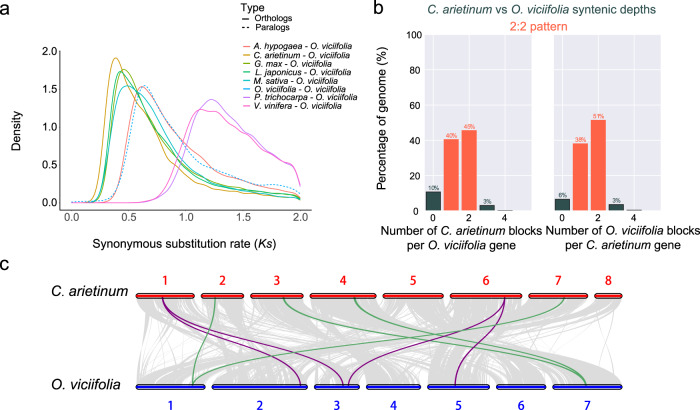
WGD events and karyotype evolution of *O. viciifolia* and other species. **a** Distribution of synonymous substitution rates (Ks) of homologous gene pairs between *O. viciifolia* and other species. **b** Syntenic depths between *O. viciifolia* and *C. arietinum*. **c** Chromosome-scale synteny analysis between *O. viciifolia* and *C. arietinum*.

We investigated the history of genome evolution in *O. viciifolia* based on the synonymous substitution rate (*Ks*), with special interest in whole-genome duplication (WGD) and whole-genome triplication (WGT) events, and found that the distribution of *Ks* values confirmed the Papilionoideae whole-genome duplication (PWGD) for legume species (*A. hypogaea*, *C. arietinum*, *G. max*, *L. japonicus*, and *M. sativa*) and the γ polyploidization event (γ-WGT) for nonlegume species (*P. trichocarpa* and *V. vinifera*) (Fig. 3a, Supplementary Fig. 7 and Supplementary Data 3). The *Ks* peak at ~0.5–1.0 based on paralogous genes in five species indicated the presence of the shared PWGD, and another peak at ~0–0.5 in *G. max* and *A. hypogaea* (Supplementary Fig. 7) corresponded to one recent additional WGD event, which was consistent with previous reports^45,46^. We found that the orthologous genes that experienced the PWGD event were mainly enriched in KEGG pathways related to signal transduction (Supplementary Table 19) and enriched in biological processes related to signal transduction, response to environment, response to hormones, and development of different organs in GO analysis (Supplementary Table 20).

Comparative genomics revealed a significant percentage of orthologous genes with a 2:2 ratio between *O. viciifolia* and the other four species, namely, *C. arietinum*, *M. sativa*, *M. truncatula*, and *L. japonicus* (Fig. 3b, Supplementary Fig. 8 and Supplementary Data 4), which suggested that these Fabales species all experienced a single WGD event, i.e., the PWGD event (Fig. 3c). Furthermore, a ratio of 4:2 was detected between *A. hypogaea* and *O. viciifolia*, which further confirmed the PWGD event in both species and one more WGD event in *A. hypogaea* (Supplementary Fig. 8d).

### Genome synteny and chromosomal arrangements

Genome collinearity of *O. viciifolia* was explored among the selected species *C. arietinum*, *M. sativa*, *M. truncatula*, *P. sativum*, *L. japonicus*, *G. max*, *V. radiata*, *C. cajan*, *A. hypogaea*, *A. duranensis*, *P. persica*, *P. trichocarpa*, and *V. vinifera* (Supplementary Fig. 9), and a high level of collinearity was discovered, especially between *O. viciifolia* and some Fabales species, such as *L. japonicus*, *C. arietinum*, *P. sativum*, *M. sativa*, and *M. truncatula*, which are all closer than *P. persica*, *P. trichocarpa*, and *V. vinifera* to *O. viciifolia* in the phylogenetic tree. Several chromosomes in *C. arietinum* showed a high level of collinearity with *O. viciifolia*, such as OvChr1 vs. CaChr7, OvChr4 vs. CaChr3, OvChr5 vs. CaChr6, OvChr6 vs. CaChr5, and OvChr7 vs. CaChr4 (Supplementary Fig. 9a). Meanwhile, chromosomes with high similarity and collinear relationships were found between *O. viciifolia* and *M. sativa*, including OvChr1 vs. MsChr8, OvChr4 vs. MsChr7, OvChr5 vs. MsChr4, OvChr6 vs. MsChr3, and OvChr7 vs. MsChr1 (Supplementary Fig. 9b). The *O. viciifolia* chromosomes OvChr1, OvChr4, OvChr5, OvChr6, and OvChr7 were relatively conserved and showed the same ancestral chromosomal structure, as observed in comparison with *C. arietinum*, *P. sativum*, *M. truncatula*, and *M. sativa* (Supplementary Fig. 9a–d). However, they still carried significant variations and chromosomal rearrangements compared to the ancestral eudicot karyotype (AEK), as the seven ancestral chromosomes were still retained in *V. vinifera* with only the γ-WGT event, and collinearity was poor between *V. vinifera* and *O. viciifolia* (Supplementary Fig. 9m).

**Fig. 4:**
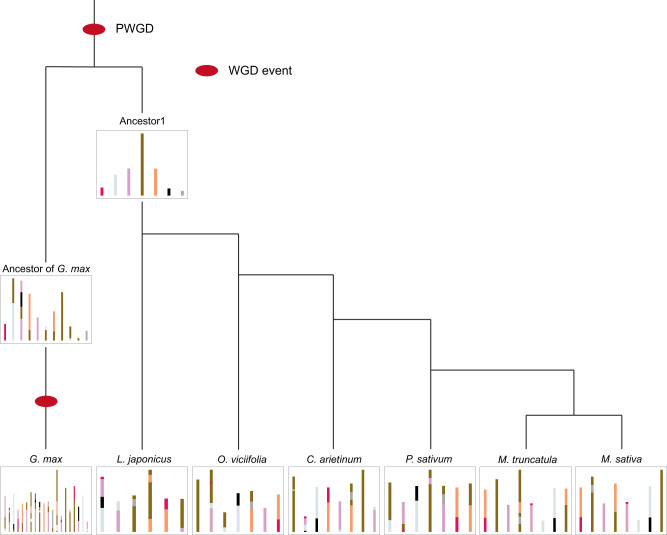
Karyotype evolutionary history of selected leguminous species. Ancestor1 was the common ancestor of selected Hologalegina plants, and chromosomes are highlighted in different colors.

Further karyotype evolutionary history analysis was performed to determine the ancestral Hologalegina karyotype (AHK) for *O. viciifolia* and six other Hologalegina plant species, *L. japonicus*, *O. viciifolia*, *C. arietinum*, *P. sativum*, *M. truncatula*, *M. sativa*, and *G. max*. The results showed that there were seven ancestral chromosomes in Ancestor1 (Fig. 4). The AHK for Ancestor1 experienced an enormous number of chromosomal rearrangement events and evolved into the modern Hologalegina karyotypes. It was inferred that Ancestor1 experienced 16 fission and 16 fusion events and evolved into the modern *O. viciifolia* karyotype (Supplementary Table 21). Chromosomal conservation was observed and confirmed between *O. viciifolia* and Ancestor1. Conservation could also be found in the complete synteny block between *O. viciifolia* and *C. arietinum* (Supplementary Fig. 9a).

We rebuilt the karyotype evolutionary events for the inferred intermediate ancestral nodes (Supplementary Fig. 10). The results showed that chromosomal breakage of Ancestor1 with 7 chromosomes generated up to 8 chromosomes for Ancestor2, Ancestor3, Ancestor4, and Ancestor5. Ancient *O. viciifolia* lost one chromosome, and modern *O. viciifolia* retained 7 chromosomes.

### Expression of genes in the PA biosynthesis pathway

We collected 41 germplasm lines of tetraploid *O. viciifolia* from 13 countries and regions (Supplementary Table 22) and measured PA contents for 59 individuals (Supplementary Table 23). According to the PA content, these lines were divided into three groups, including high (H), medium (M) and low (L), and nine lines were selected (3 typical independent lines with similar PA contents for each group) for transcriptome sequencing to explore the expression levels of genes involved in PA biosynthesis and transport (Supplementary Fig. 11a and Supplementary Tables 23 and 24). Our RNA-seq analysis identified 567 differentially expressed genes (DEGs) for H samples compared to L samples, 631 DEGs for H vs. M samples, and 830 DEGs for M vs. L samples. We conducted KEGG enrichment analysis to identify the differentially expressed genes in the biosynthesis pathway of PAs in sainfoin leaves with high PA content. The up- and downregulated DEGs in the pairwise comparisons of the three groups were mainly enriched in pathways of “carbon metabolism”, “biosynthesis of amino acids”, “phagosome”, etc. (Supplementary Fig. 11b–d).

Weighted gene coexpression network analysis (WGCNA) was used to generate 9 gene modules after merging similar gene expression modules (Supplementary Fig. 12a). The correlation relationships between traits and gene modules showed that the MEblack module exhibited the highest correlation with PA content. In this module, LeOno02aG0012600, which was annotated as an anthocyanidin synthase (ANS) gene, was the hub gene with 37 other genes in the coexpression network (Supplementary Figs. 12b, 13 and Supplementary Table 25), suggesting a key role of ANS in PA biosynthesis.

### Expansion of genes involved in PA biosynthesis

**Fig. 5:**
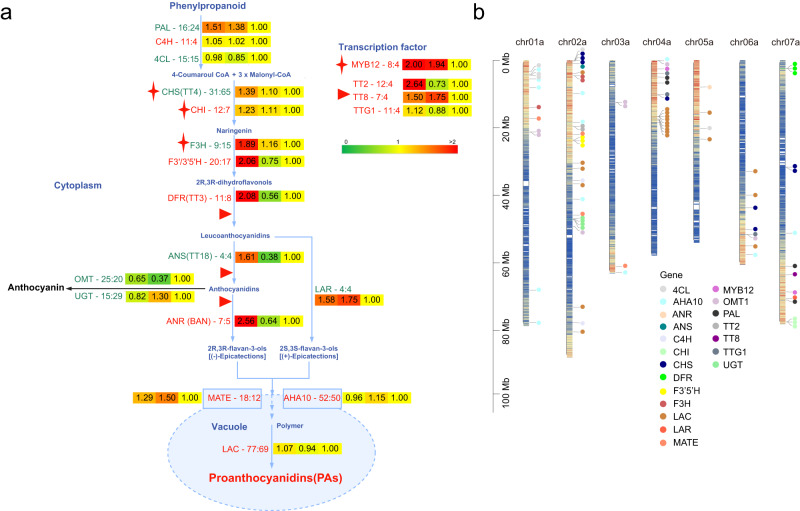
Expansion and expression of genes involved in PA biosynthesis. **a** Heatmaps showing the fold changes in the expression levels of genes in the PA biosynthesis pathway among the three groups of H, M, and L (the left is for H, the center is for M, and the right is for L). The numbers in the heatmap represent the fold change values, which were calculated with the gene expression in Groups H and M compared with their counterparts in Group L. The gene copy numbers are shown following the enzymes and TFs in the whole biosynthesis pathway in a comparison of *O. viciifolia* and *M. sativa* “XinjiangDaye” (the left is for *O. viciifolia*, and the right is for *M. sativa*). The genes are highlighted in red if the copy number is higher in *O. viciifolia* than in *M. sativa*, and otherwise in green. L-phenylalanine ammonia-lyase, PAL; cinnamate 4-hydroxylase, C4H; 4-coumarate coenzyme A ligase, 4CL; chalcone isomerase, CHI; chalcone synthase, CHS; flavanone-3-hydroxylase, F3H; “flavonoid 3’ hydroxylase”/“flavonoid 3'5’ hydroxylase”, F3’/3’5’H; dihydroflavonol 4-reductase, DFR; anthocyanidin reductase, ANR; leucoanthocyanidin reductase, LAR; multidrug and toxic compound extrusion-type transporter, MATE; plasma membrane H^+^-ATPase, AHA10; laccase-like flavonoid oxidase, LAC. **b** Homologous genes involved in the PA biosynthesis pathway plotted on the seven chromosomes of haplotype A of *O. viciifolia*.

Based on the published literature, we summarized the whole pathway of PA biosynthesis, which started from phenylalanine to PAs (Fig. 5a), and multiple regulators, including synthesis enzymes and TFs, functioning in key steps of the PA biosynthesis pathway. We collected the sequences of genes involved in the pathway of PA biosynthesis in *A. thaliana*, *V. vinifera* and *G. max* (Supplementary Table 26) and determined the orthologous genes and their copy numbers in *O. viciifolia* in comparison with the other four Fabales species, *M. sativa* cultivar XinJiangDaYe”, *Lotus japonicus* “MG20”, *Medicago truncatula* “A17”, and *Glycine max* “Wm82”, based on all the annotated proteins downloaded from the NCBI database. We found that the autotetraploid *O. viciifolia* and *M. sativa* gained more copy numbers than the other three diploid Fabales species (Fig. 5a and Supplementary Fig. 14). Meanwhile, compared to autotetraploid *M. sativa*, we also revealed gene expansion and copy number gain in *O. viciifolia*, as more gene copy numbers were found, for a total of 13 related regulators in the PA biosynthesis pathway, namely, C4H, CHI, F3’/3'5’H, DFR, ANR, MATE, AHA10, LAC, MYB12, TT2, TT8, and TTG1, in *O. viciifolia* (highlighted in red in Fig. 5a). It is worth noting that the copy number of genes encoding O-methyltransferase (OMT), a key enzyme for anthocyanin biosynthesis, was significantly higher in *O. viciifolia* than in the other diploid Fabales species.

Furthermore, we plotted the homologous genes of the PA biosynthesis pathway onto the chromosomes (Fig. 5b and Supplementary Fig. 15) and found many tandemly repeated genes on the chromosomes, such as CHS genes (LeOno02aG0003100, LeOno02aG0003200, LeOno02aG0003300) on chr02a and LAC genes (LeOno04aG0201900, LeOno04aG0202000, LeOno04aG0202100) on chr04a (Fig. 5b). The intensive tandem repeat genes belonged to the paralogous genes, and similar results were found in the other three haplotypes (Supplementary Fig. 15). Based on the results, we inferred that one of the reasons for the enrichment of PAs in sainfoin may be that the generation of tandem repeats contributed greatly to the enrichment of PAs in terms of gene dosage effects.

In fact, it is known that PA content is significantly higher in *O. viciifolia* than in *M. sativa*, although both of these two species are important leguminous forage crops and autotetraploids^47,48^. To check the effects of gene expansion, RNA-seq and qRT‒PCR were performed to uncover the expression levels of genes involved in the PA biosynthesis pathway in the leaves of *O. viciifolia* and *M. sativa*. The RNA-seq results showed that genes were mostly more highly expressed in the three *O. viciifolia* groups with high, medium, and low PA contents than in alfalfa cultivar “Zhongmu No. 1” (Supplementary Table 27), with GADPH, CYP, and EF1a as internal reference genes. qRT‒PCR further confirmed the significantly higher expression levels of PAL, ANR, ANS, F3’5’H, and F3H in the leaves of *O. viciifolia* compared to *M. sativa* (Supplementary Fig. 16). These results suggest the contribution of gene expansion to the expression of genes involved in PA biosynthesis in *O. viciifolia*.

Furthermore, we found increased expression of genes involved in PA biosynthesis in the *O. viciifolia* lines with high PA content by using RNA-seq analysis of three *O. viciifolia* groups (H, M, and L). The expression levels of genes involved in the PA biosynthesis pathway were mostly all significantly increased in Group H (left in the heatmap of fold changes of gene expression) compared to M (middle) and L (right) (Fig. 5a). In addition, the genes *OMT* and *UGT*, which are responsible for anthocyanin biosynthesis, were downregulated in Group H, which implied that the biosynthesis pipeline predominantly involved the production of PA, rather than anthocyanin, in the leaves of *O. viciifolia* lines with high PA content. Taken together, these results indicate that gene expansion is the key driving force for the enhancement of PA production in *O. viciifolia*.

## Discussion

*O. viciifolia* is a widely cultivated leguminous forage with high contents of both protein and PA, and the *O. viciifolia* genome assembly, as the one for Hedysareae plants, is valuable for understanding the genome evolution and phylogenetic relationships of Fabales species, for which >10 genome assemblies are available^49^. However, it is a major challenge to assemble the genome of *O. viciifolia* since it is autotetraploid and cross-pollinated. In our study, we applied next-generation technology and Hi-C technology to obtain a relatively complete tetraploid genome for sainfoin.

It is valuable but difficult to assemble the whole genome, including four haplotypes, of autotetraploids to obtain whole-genome information, which mainly includes unique genes existing in different haplotypes. The reason for the difficulty is the lack of ability to identify and separate the extremely similar haplotypes^50^. Currently, with the help of new sequencing technology, especially third-generation sequencing technology for long reads and high-throughput chromatin conformation capture technology, a highly prominent number of complete genomes of autotetraploid plants have been assembled and reported^34,36,51–54^. In summary, there are three main strategies used to assemble the four haplotypes of autotetraploid genomes. One of them is to assemble one haplotype of the autotetraploid, such as *M. sativa* “Zhongmu No. 1”^32^, but the disadvantage is that many genes might be lost because of the mixed nature of the four haplotypes under cross-pollination. The second strategy is to assemble the four haplotypes of autotetraploids based on third-generation sequencing and Hi-C data, such as the recent allele-aware genomes of autotetraploid “Zhongmu No. 4”^36^, autotetraploid potato “Q9”^53^, tetraploid highbush blueberry cultivar “Draper”^52^, and autopolyploid sugarcane “Np-X”^54^. The third strategy of autotetraploid assembly is to use additional methods to improve autotetraploid genome phasing, such as linkage grouping in a population of hybrid progenies^51^ or single-gamete sequencing^43^. Based on the four haplotypes of autotetraploids, researchers discovered many more genes and structural rearrangements, and the results markedly promoted the development of genomics and functional genomics.

In this study, we used the second strategy and successfully assembled four haplotypes of the *O. viciifolia* chromosome-level genome. The contig N50 of 10.41 Mb was much longer than those of recently reported leguminous species genome assemblies, such as those of *A. sinicus* and *M. sativa*^33,36^. BUSCO analysis revealed 89.1%–90.4% complete genes in the four haplotypes and 91.3% in the whole genome upon genome assembly (Supplementary Tables 5 and 10). In addition, the alignment rates of RNA-seq data were 80.86%–86.22% in the four haplotypes and 90.13%–93.30% for the whole genome (Supplementary Table 24). This study provides a complete genome resource for *O. viciifolia* to search for copies of genes involved in PA biosynthesis and will promote more functional genomic studies in the future.

PAs have prominent effects on fruit flavor, forage quality, and plant defence^55–57^ and could reduce rumen bloat disease and methane emission in ruminants^58^. PAs are synthesized by a long pipeline that involves several key enzymes and TFs (Fig. 5a) in plants^27,28,59^. Gene expansion is a key strategy employed by plants for environmental adaptation under the enhancement of secondary metabolite production due to the quantitative dose effects and gene expression regulation network. Copy numbers of PAL, 4CL, and CHS genes that were key in the flavonoid pathway were higher in the genome of *Cenchrus purpureus* with more anthocyanidin than in the genomes of *C. americanus* and *Setaria italica* with less anthocyanidin^60^. Tandem repeats of CHS genes, which are related to the first rate-limiting enzyme in flavonoid biosynthesis, were found in *M. truncatula*^33^. In our study, we confirmed that the enrichment of PAs in *O. viciifolia* was significantly contributed by gene expansion and expression upregulation. We revealed significant expansion of genes involved in PA biosynthesis in *O. viciifolia* compared with the closely related autotetraploid species *M. sativa*, although *M. sativa* has a large genome size of 3157 Mb and 164,632 protein-coding genes^34^. The upregulated expression of these genes further resulted in the enhancement of PA content in *O. viciifolia*. More than half of all plant species experienced polyploidy, including autopolyploidy and allopolyploidy^61^. It was shown that in allotetraploid elephant grass (*Cenchrus purpureus* Schumach.) with high anthocyanidin content, a gene expansion strategy was also adopted to enhance the biosynthesis of anthocyanidins and flavonoids^60^. Consequently, the induced autopolyploids exhibited enhanced resistance to biotic and abiotic stresses, and secondary metabolite production might be considerably increased^62^. Cases were reported that confirmed this link between the enhancement of secondary metabolite production and gene copy number increase. For example, cichoric acid^63^, caffeic acid^64^, and flavonoids^65^ were found at higher levels in autotetraploid plants. In addition, in our case, the downregulated expression of both OMT and UGT in the leaves of *O. viciifolia* was one key reason that anthocyanidins were converted into PAs rather than anthocyanins.

A recent study revealed that *MtGSTF7*, a homologous gene of *AtTT19*, in *Medicago truncatula* played an important role in the accumulation and translocation of both anthocyanin and PAs^66^. However, the homologous genes were not expressed in *O. viciifolia* (i.e., LeOno02aG0476700, LeOno02bG0495900, LeOno02cG0475100, and LeOno02dG0404000) and *M. sativa* leaves. Therefore, we suspected that there would be other genes involved in the accumulation and translocation of PAs, which could be discovered in the future. Meanwhile, PAs are enriched in different tissues in various plant species. For example, *O. viciifolia* and *L. japonicus* can enrich PAs in all organs, but PAs are mainly enriched in the seed coat of *M. sativa* and flowers of *T. pratense*^15^. Phagosomes, involved in a cellular homeostatic process, were significantly enriched in the lines of *O. viciifolia* with a high PA content (Supplementary Fig. 11b). Plants enriched in PAs synthesize monomeric flavonoids, such as flavan-3-ol and epi-flavan-3-ol, in the cytoplasm and transport them into the vacuole to synthesize PAs in the final polymerization step^15^. Some researchers found that autophagy involved trafficking of anthocyanin from the cytoplasm to vacuoles, as anthocyanin content was reduced in autophagy-deficient plants^67–69^. Overexpression of MdATG18a enhanced autophagy activity and improved anthocyanin content in apple^70^. The phagosome-mediated autophagosome transport mechanism of monomeric flavonoids might play a key role in PA biosynthesis in *O. viciifolia*. Autophagy and autophagosomes control peroxisome quality and are involved in the degradation of peroxisomes^71^, which is consistent with the downregulation of genes related to peroxisomes (Supplementary Fig. 11b). To date, several genes have been reported to play a role in PA biosynthesis^15,72,73^, among which ANS is vital in the production pathway of flavonoids. ANS is a key enzyme that catalyses leucoanthocyanidins into anthocyanidins, which serve as substrates for the production of anthocyanins and PAs^15^. Our coexpression network results showed a strong correlation between PA content and the MEblack module and further identified ANS (LeOno02aG0012600) as the hub gene in the gene regulatory network for the PA biosynthesis pathway of *O. viciifolia* leaves, which was in accordance with the results found in *Tetrastigma hemsleyanum*^74^. The genes in the MEblack module are valuable for further investigation of their roles in PA biosynthesis.

## Materials and methods

### Plant materials and preparation for sequencing

We chose one robust plant of common sainfoin that was grown in the greenhouse of CAU (China Agricultural University) and cut its leaves before immediately processing them in liquid nitrogen. Genomic DNA was extracted from the young leaves using a modified CTAB method previously described^75^. An Agilent 2100 instrument and electrophoresis were used to assess DNA quality. Whole-genome sequencing was performed by combining the Oxford Nanopore technology (ONT, ~109.89×) and Illumina (~96.15×) approaches (Supplementary Table 1). Young leaf tissue was used for Hi-C library construction based on HindIII digestion. The Hi-C library was sequenced on an Illumina HiSeq 2500 platform in paired-end 150 bp mode (~164.84×). All sequencing was conducted by BioMarker Technologies Company (Beijing, China).

A total of 59 individuals of 41 germplasm lines of *O. viciifolia* were used to determine the PA content (Supplementary Tables 22 and 23). They were grouped into three groups, which corresponded to high, medium, and low levels of PA. Three independent representative individuals from each group were selected for transcriptome sequencing of leaf tissue. RNA extraction and quality assessment were performed according to a previous method^76^. We also downloaded the transcriptome data of sainfoin from the National Center for Biotechnology Information (https://www.ncbi.nlm.nih.gov) to annotate our genome assembly (Supplementary Table 8).

### Genome size estimation

We estimated genome size roughly through the two strategies of *K*-mer analysis based on Illumina short reads of genomic DNA and flow cytometry, for which *Medicago truncatula* (cultivar “A17”), *Panicum virgatum* (cultivar “Alamo”), and *Zea mays* (B73) were used as internal references. Clean short reads were subjected to counting of each 17-mer using Jellyfish 2.0 (www.genome.umd.edu/jellyfish.html). The results of Jellyfish were input into GenomeScope2 (http://qb.cshl.edu/genomescope/genomescope2.0) to estimate genome size and heterozygosity (Supplementary Fig. 1).

### Genome assembly

ONT raw reads were corrected by using NextDenovo v2.5.0 (parameter: correction_options = -b) and initially assembled into contigs using Flye v2.9 (parameter: --keep-haplotypes --iterations 2)^77^, assembling contigs of four haplotypes as completely as possible. The contigs were polished with Illumina short reads to remove nucleotide errors using Pilon47 v1.24^78^. There were small-scale collapses and redundancy in the four haplotypes, and some contigs from different haplotypes were incorrectly linked together. Haplotypic chromosome-level assembly was accomplished using the Hi-C technique. First, following a previously described pipeline^51^, we aligned Hi-C reads to the initial genome assembly using Juicer v1.6^79^, and Hi-C-assisted chromosomal assembly was conducted by using 3D-DNA v180922^80^ to correct the majority of the assembly errors. Then, based on the interaction of Hi-C data, manual inspection and adjustment, including adjusting the boundary of chromosomal segmentation and correcting visible errors as much as possible, were performed to generate the final allele-aware chromosomal assembly in Juicebox v2.14.00^81^. It is widely accepted that the interaction among reads with shorter distances is stronger than that among reads with long distances in Hi-C analysis. The assembly was finally generated and grouped into 28 pseudochromosomes, without significant misassembly. However, we still obtained some collapsed contigs. After manual examination, gaps were filled by using LR_Gapcloser^82^ based on ONT long reads. The final genome assembly was generated after three rounds of polishing by using Pilon47 v1.24.

We mapped the Illumina short reads, ONT long reads, and RNA-Seq reads to the genome assembly with BWA-MEM v0.7.15^83^, Minimap2 v2.24^84^, and HISAT2 v2.2.1^85–87^, respectively. The read mapping rate was calculated to assess assembly integrity. BUSCO v5.2.2^88^ was used to evaluate the quality and completeness of the assembly (parameters: -m genome --augustus) based on embryophyte_odb9. Based on the Illumina short reads, we further assessed the whole genome and four haplotypes of *O. viciifolia* by using Merqury v1.3 and Meryl v1.4^89^. And based on the ONT long reads, we used Inspector-v1.2 to identify the regions with collapse and switch error in the whole genome^90^.

### Repeat identification and gene annotation

We identified transposable elements with the ab initio method by EDTA v2.0.0 (parameter: --sensitive 1 --anno 1)^91^ and used RepeatMasker (http://www.repeatmasker.org/RepeatMasker/) to predict repeat sequences. We identified tRNAs by using tRNAScan-SE v2.0.9^92^, rRNA by using Barrnap (https://github.com/tseemann/barrnap), and noncoding RNA with RfamScan^93^.

The combined strategies of ab initio prediction and evidence- and homolog-based searching methods were used for gene modeling. The transcript evidence came from the two transcriptome assemblies. The first de novo transcriptome assembly was conducted based on the high-quality RNA-seq reads using Trinity v2.13.2^94^, and the second transcriptome assembly was accomplished by using HISAT2 alignment and genome-guided Trinity assembly. In total, 326,841 transcript sequences were obtained after merging these two transcriptome assemblies and removing redundant sequences by using CD-HIT v4.6 (95% identity and 95% coverage)^95^. Based on the above transcript sequences, ab initio gene predictions were produced by PASA v2.4.1^96^ and AUGUSTUS v3.4.0^97^ from the genome assembly and optimized for five rounds by using AUGUSTUS. In addition, we also ran the MAKER2 pipeline^98^ to build gene models. The alignments from 326,841 transcripts obtained by using BLASTN and 200,995 homologous proteins from *Glycine max*, *Medicago truncatula*, *Cicer arietinum*, *Vitis vinifera*, *Arabidopsis thaliana*, and *Oryza sativa* obtained by using BLASTP were run against the repeat-masked genome assembly and manipulated with Exonerate v2.4.0^99^. The ab initio gene predictions from AUGUSTUS and evidence- and homolog-based gene annotations were merged using the maker2eval script packaged with MAKER2. To increase accuracy and completeness^100^, the gene models from MAKER2 and PASA were integrated and combined into the final gene models using EVidenceModeler (EVM) v1.1.1^101^.

For gene functional annotation, the predicted gene models were subjected to homology searches against the following databases: Kyoto Encyclopedia of Genes and Genomes (KEGG), Gene Ontology (GO), and Clusters of Orthologous Groups of proteins (KOG/COG/eggNOG) by using eggNOG-mapper v2.1.6^102^; NCBI nonredundant protein sequences (NR) and Swiss-Prot by using DIAMOND v2.0.14^103^; and Pfam by using InterProScan v5.54-87.0^104^.

### Gene family and phylogenetic analysis

We selected 14 legume species, i.e., *M. sativa*, *M. truncatula*, *M. runthenica*, *T. subterraneum*, *T. pratense*, *P. sativum*, *C. arietinum*, *L. japonicus*, *G. max*, *V. radiata*, *C. cajan*, *L. angustifolius*, *A. hypogaea*, and *A. duranensis*, and four other outgroup species (*P. persica*, *A. thaliana*, *P. trichocarpa*, and *V. vinifera*), and downloaded their protein sequences from the NCBI. Protein sequences of the *O. viciifolia* genome assembly and these downloaded genome assemblies were used to identify orthologues by using OrthoFinder2 v2.5.4 with a parameter of “-M msa”^105^. In total, 448 single-copy proteins were obtained, and their sequences were concatenated to build a phylogenetic tree with the maximum likelihood method by using IQTREE v2.1.6^106^ based on the JTT + F + R5 model and 1000 bootstraps. This ML tree and the 448 single-copy orthogroups were subjected to divergence time estimation by the MCMCTree program in the PAML package v4.10.3^107^. Three time-calibration points were selected, including *M. sativa* vs. *O. viciifolia* 15–91 million years ago (Mya); *A. hypogaea* vs. *O. viciifolia* 25.9–120 million years ago (Mya); and *V. vinifera* vs. *O. viciifolia* 107–135 Mya, which are available on the Timetree website (http://timetree.org/). By determining the numbers of gene families and genes with OrthoFinder2, we obtained and compared statistical information on the gene families of *O. viciifolia*, *M. sativa*, *L. japonicus*, and *C. arietinum*.

### Genome evolutionary analysis

Based on orthologous genes, genome collinearity analysis among species and between haplotypes was performed by using MCScanX and viewed in JCVI^108^. Based on syntenic or homologous gene pairs obtained from MCScanX, *K*a, *K*s, and *K*a/*K*s values were calculated by using TBtools^109^, and the *K*s distribution was used for inferring genome palaeopolyploidization events and occurrence time estimation. LTRs were identified by LTR_harvest V1.6.2^110^ (parameters: -similar 90 -vic 10 -seed 20 -seqids yes -minlenltr 100 -maxlenltr 7000 -mintsd 4 -maxtsd 6 -motif TGCA -motifmis 1), and LTR insertion time was calculated by LTR_retriever V2.9.0^111^ (parameters: -u 7e-9).

We used CAFE 5^112,113^ to identify the expanded, contracted, and rapidly evolved gene families based on the phylogenetic tree and 27,749 homologous gene families from OrthoFinder2.

### Ancestral karyotype evolution

We selected seven leguminous species, namely, *O. viciifolia*, *M. sativa*, *M. truncatula*, *P. sativum*, *C. arietinum*, and *L. japonicus*, with *G. max* as an outgroup species. Based on the published pipeline (https://github.com/xjtu-omics/processDrimm) (Gao et al. 2022)^114^, orthogroups of complete homologous gene sequences were identified by using Orthofinder v2.5.5 (https://github.com/davidemms/OrthoFinder), and the nonoverlapping syntenic blocks were built by using Drimm-Synteny (https://github.com/xjtu-omics/processDrimm/tree/master/drimm). The longest common subsequence (LCS) algorithm was used to retrieve the gene sequences for each block and their copy number in each species. The ancestors’ genomes were rebuilt based on the gene sequences by using IAGS scripts (https://github.com/xjtu-omics/IAGS).

### Expansion of genes involved in PA biosynthesis between *O. viciifolia* and *M. sativa*

We downloaded the reference protein sequences of *A. thaliana*, *V. vinifera*, and *G. max* (Supplementary Table 25) and identified the copy numbers of genes involved in the PA biosynthesis pathway based on the protein sequences of *O. viciifolia*, *M. sativa* cultivar “XinjiangDaye” (https://figshare.com/projects/whole_genome_sequencing_and_assembly_of_Medicago_sativa/66380), *Lotus japonicus* (GenBank accession GCA_000181115.2), *Medicago truncatula* (GenBank accession GCF_003473485.1), and *Glycine max* (GenBank accession GCF_000004515.6). The cut-offs of BLASTP were set with an E-value of less than 1e^−150^ and identity of >50%. The homologous genes of *O. viciifolia* were plotted on the chromosomes by using TBtools v1.113.

### Transcriptome analysis for gene expression in leaves

RNA-seq data from the leaves of *M. sativa* (NCBI accession PRJNA795295) and three groups of *O. viciifolia* lines that showed high (Group H), medium (Group M), and low (Group L) PA contents were analyzed based on a trimming pipeline in Trimmomatic v0.39^115^, mapping by HISAT2 v2.2.1, and determining gene expression FPKM values by StringTie v2.2.0^116^. Significantly differentially expressed genes were determined by the R package DESeq2^117^. Principal component analysis (PCA) and visualization were conducted by using the R functions prcomp() and ggbiplot(). KEGG enrichment was performed by using the R package “clusterProfiler”.

In coexpression network analysis, gene modules were generated, and their correlation with PA content and other phenotypic traits was analyzed by using the R package “WGCNA”. The expression levels of all genes related to one specific regulator (including enzymes and TFs) in the PA biosynthesis pathway were summed and compared among the three groups based on the fold change values in the formula of Group H/L or M/L. The expression of genes in leaves involved in PA biosynthesis was compared between *O. viciifolia* and *M. sativa* based on the three internal reference genes GADPH, CYP, and EF1a.

For qRT‒PCR verification, total RNA was extracted from *O. viciifolia* and *M. sativa* leaves by using a Plant RNAout kit (Beijing Huayueyang Biotechnology, China), and 1 μg total RNA was used for inverse transcription and cDNA generation with a PrimeScript™ RT reagent kit with gDNA Eraser (Takara, RR047A). The qRT‒PCR experiments were performed by using 2 × ChamQ Universal SYBR qPCR Master Mix (Q711-02) and qTOWER^3^ G (Analytik Jena, Germany), with three biological replicates and GADPH/EF1a as internal control genes. The relative expression levels were determined using the 2^–ΔCt^ method. All primers were designed by Primer3 and are listed in Supplementary Table 28.

### Statistics and reproducibility

In our research, all resource data are available from the corresponding authors to ensure the reproducibility of the analysis. To advance the reproducibility, we have defined processing or sampling with a frequency more than two as replicate. There are three samples replicated at different PA content levels in the transcriptome sequencing, and two samples replicated for every species in the qRT‒PCR verification. *T*-test was used to identify the significant difference between the two samples, and linear regression analysis was preformed using the lm function of the R (v4.2.3). And Benjamini-Hochberg (BH) method was applied for *p* value correction^118^.

### Supplementary information

Supplementary Information

Description of Additional Supplementary Files

Supplementary Data 1

Supplementary Data 2

Supplementary Data 3

Supplementary Data 4

## Data Availability

All the raw data, including Nanopore long reads and Illumina short reads were uploaded to the China National Center for Bioinformation GSA (Genome Sequence Archive) database under BioProject PRJCA009631. Genome assembly and gene annotation files were available in the figshare website (10.6084/m9.figshare.24155073). The source data for Supplementary Data 1–4 is available for Figs. 1d, 2c and 3a, b respectively, while all other source data are available from S.J. and Y.Z. on reasonable request.
