# Supplementary material for: A chromosome-level genome assembly for Onobrychis viciifolia reveals gene copy number gain underlying enhanced proanthocyanidin biosynthesis
Source: Commun Biol. 2024 Jan 5;7:19. doi: 10.1038/s42003-023-05754-6 (PMC10770414; doi:10.1038/s42003-023-05754-6)
Supplement: Supplementary file 1 — Supplementary Information [file 42003_2023_5754_MOESM1_ESM.pdf]

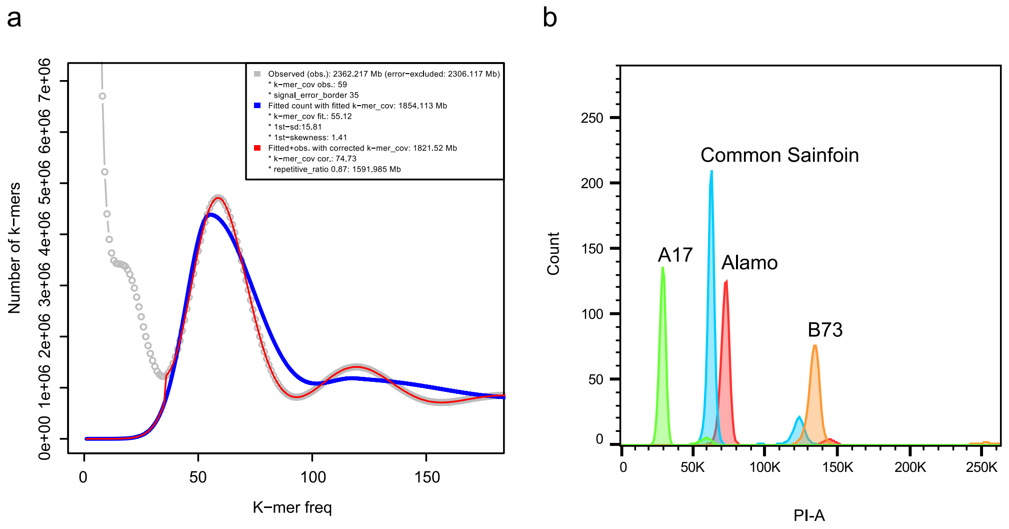

Supplementary Fig. 1 Genome size estimation of *O. viciifolia*. (a) K-mer (k=17) distribution for estimating genome size of *O. viciifolia*. (b) Flow cytometry for estimating genome size of *O. viciifolia*, with *Zea mays* B73, *Medicago truncatula* A17, and *Panicum virgatum* Alamo as internal reference plants.

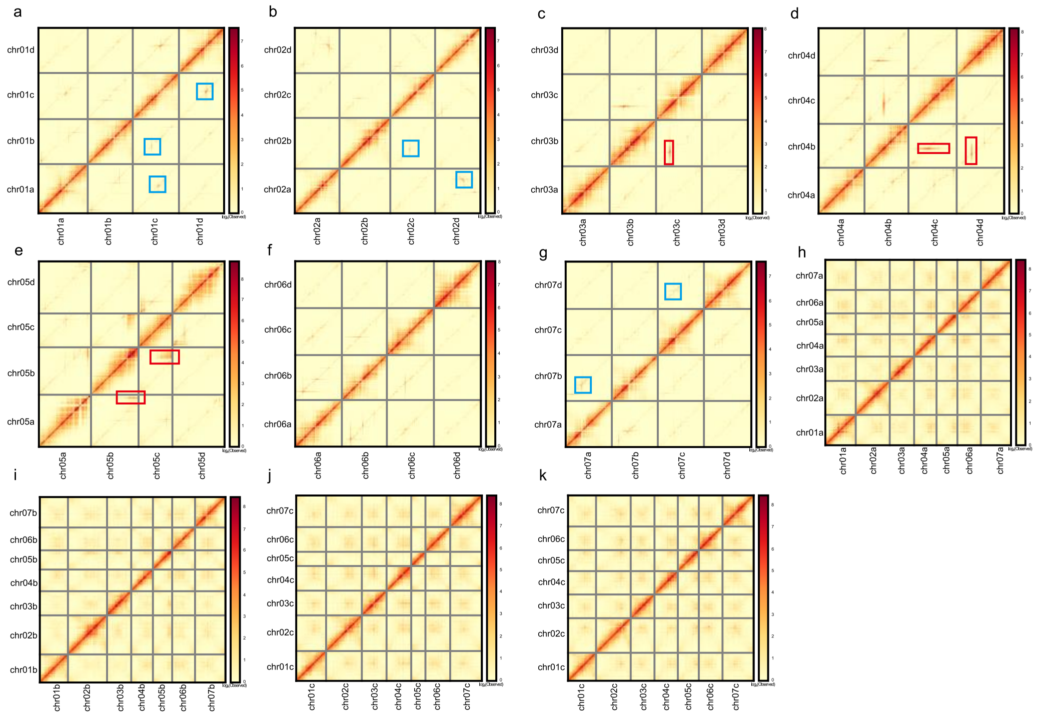

Supplementary Fig. 2 Hi-C interaction heatmap of homologous chromosomes (a-g) and haplotypes (h-k) of *O. viciifolia*. The red blocks represent the areas of collapse and the blue blocks represent the areas of switch error which can be clearly observed.

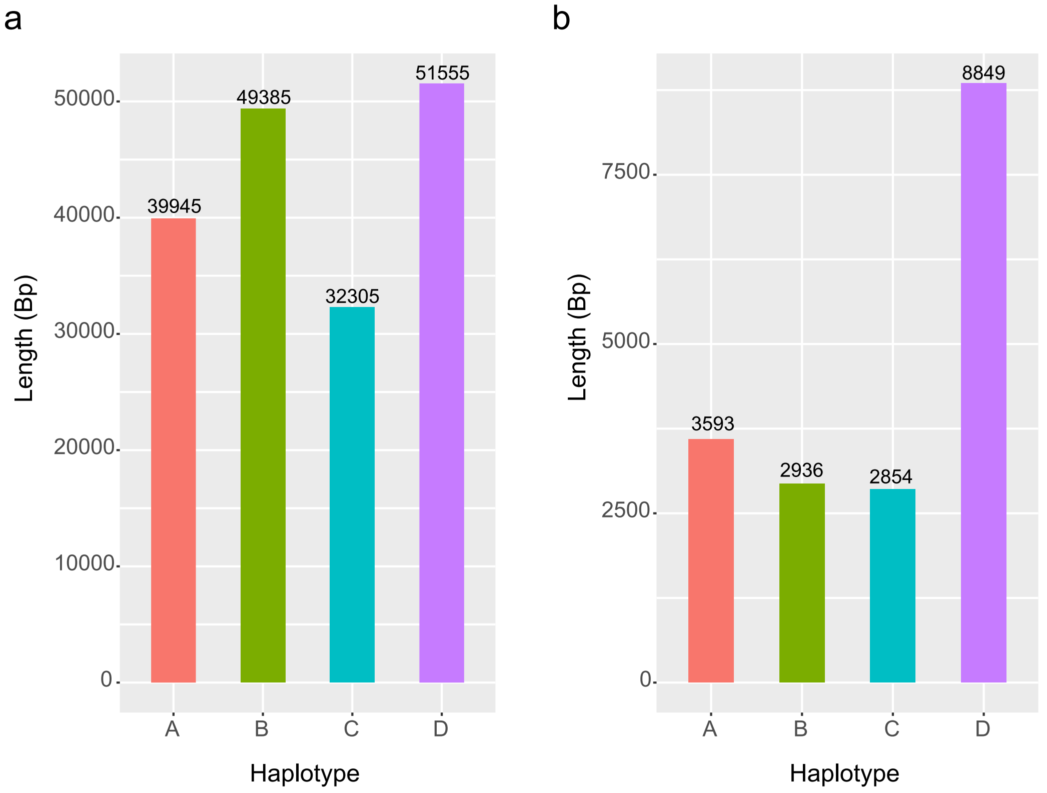

Supplementary Fig. 3 The statistics of (a) collapse and (b) switch error length in the assembled genome of *O. viciifolia*.

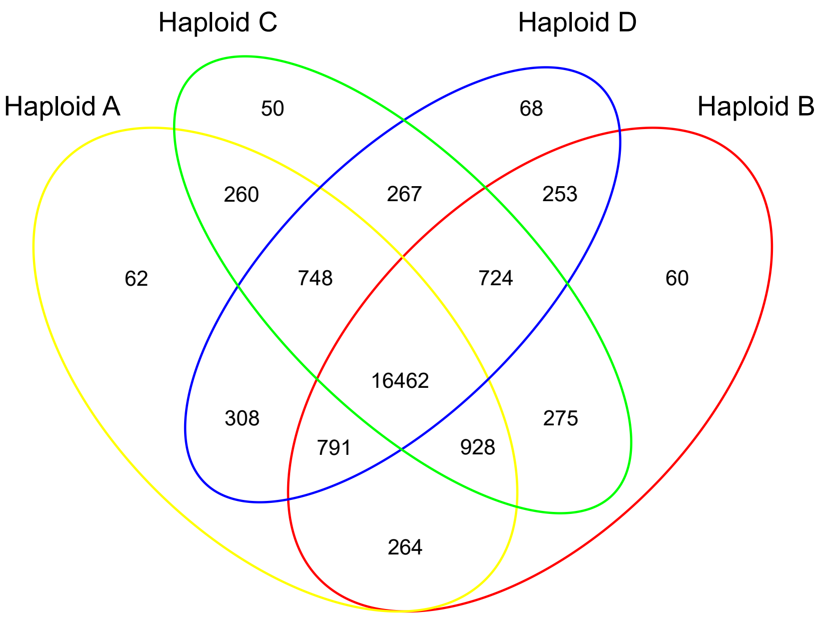

Supplementary Fig. 4 Venn plot of gene families of four haploids

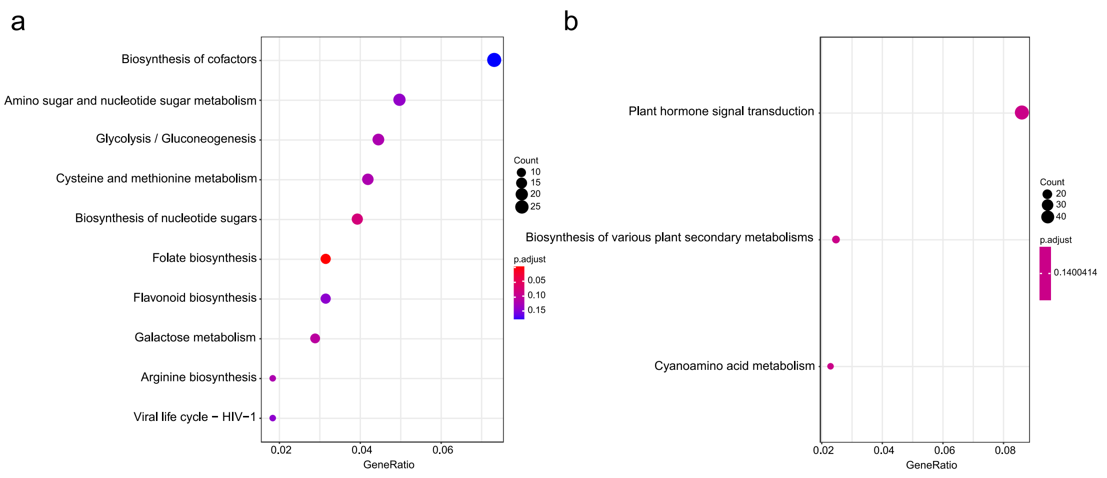

Supplementary Fig. 5 (a) KEGG enrichment of expanded gene families in *O. viciifolia*. (b) KEGG enrichment of contracted gene families in *O. viciifolia*.

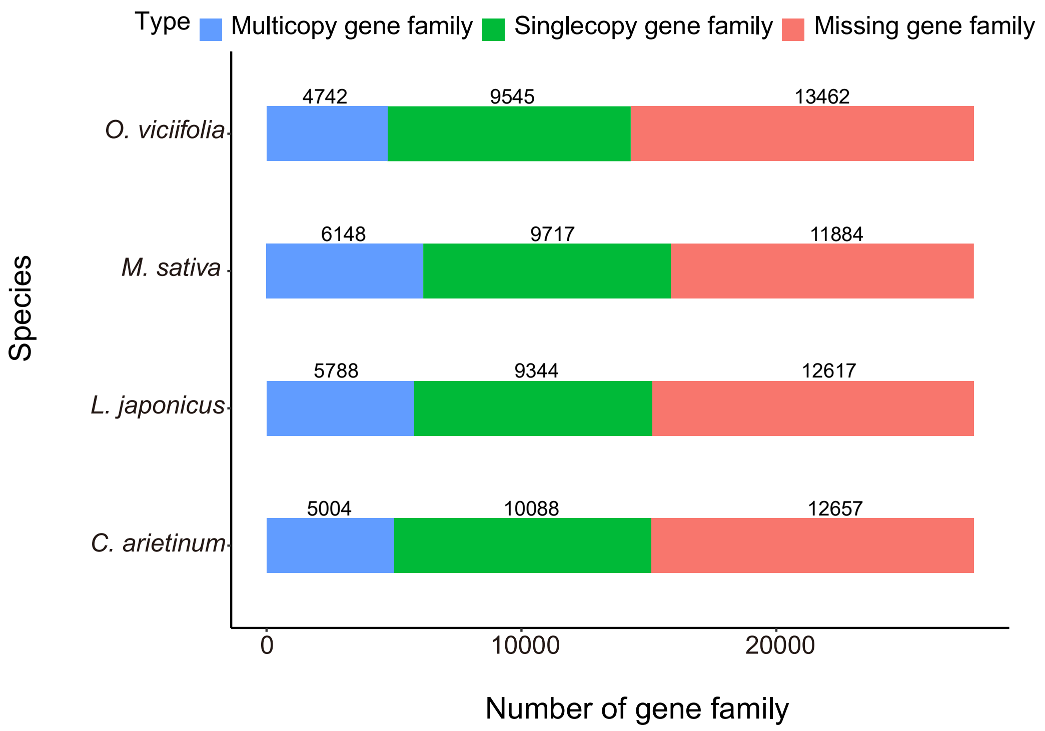

Supplementary Fig. 6 Gene families among different species. Single-copy (with only one gene in one gene family), multiple-copy, and missing gene families were shown for *C. arietinum*, *M. sativa*, *O. viciifolia*, and *L. japonicus*. The non-redundant gene families were achieved by combining the ones in the 15 Fabales species in the phylogenetic tree, and missing gene families were determined if not found in a given species.

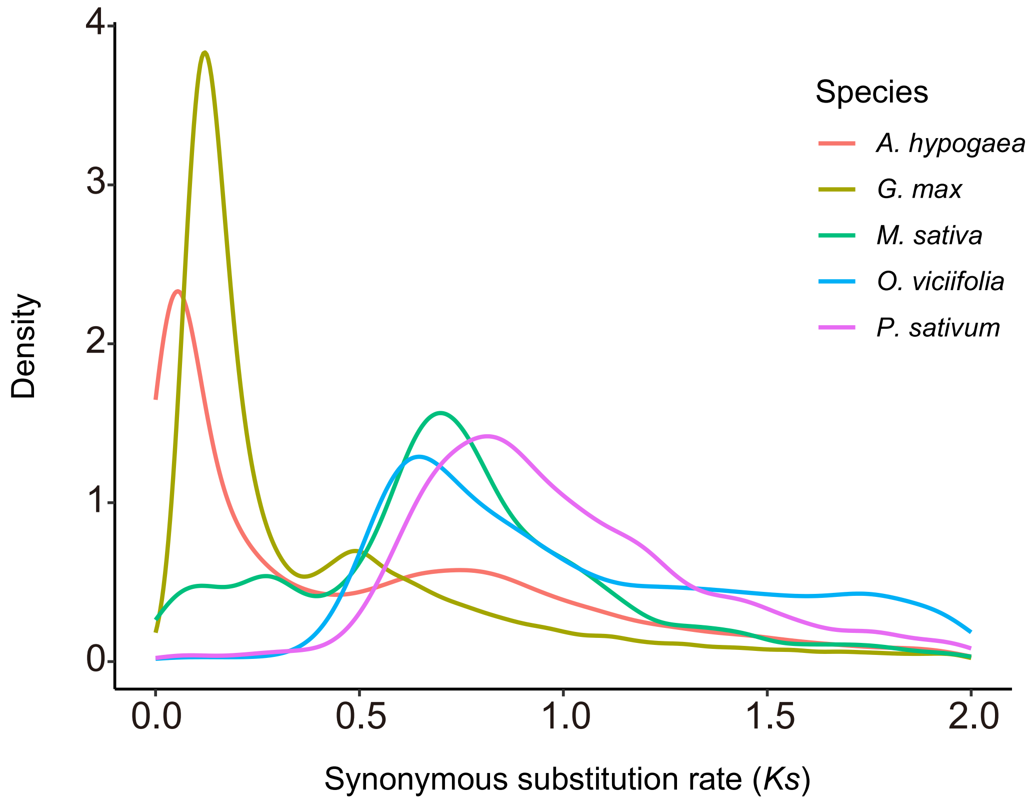

Supplementary Fig. 7 Distribution of synonymous substitution rate (*Ks*) of homologous gene pairs in *O. viciifolia* compared to other species.

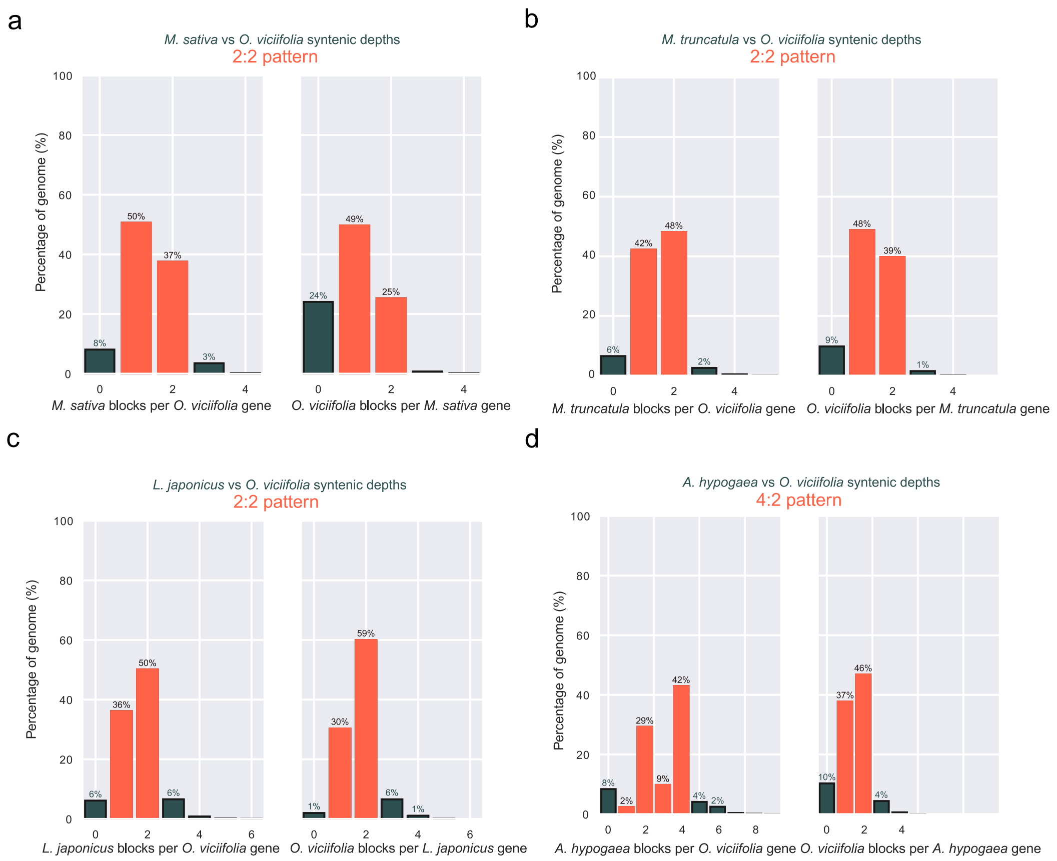

Supplementary Fig. 8 The synteny depths between *O. viciifolia* and selected species. (a) *M. sativa* vs *O. viciifolia*, (b) *M. truncatula* vs *O. viciifolia*, (c) *L. japonicus* vs *O. viciifolia*, (d) *A. hypogaea* vs *O. viciifolia*.

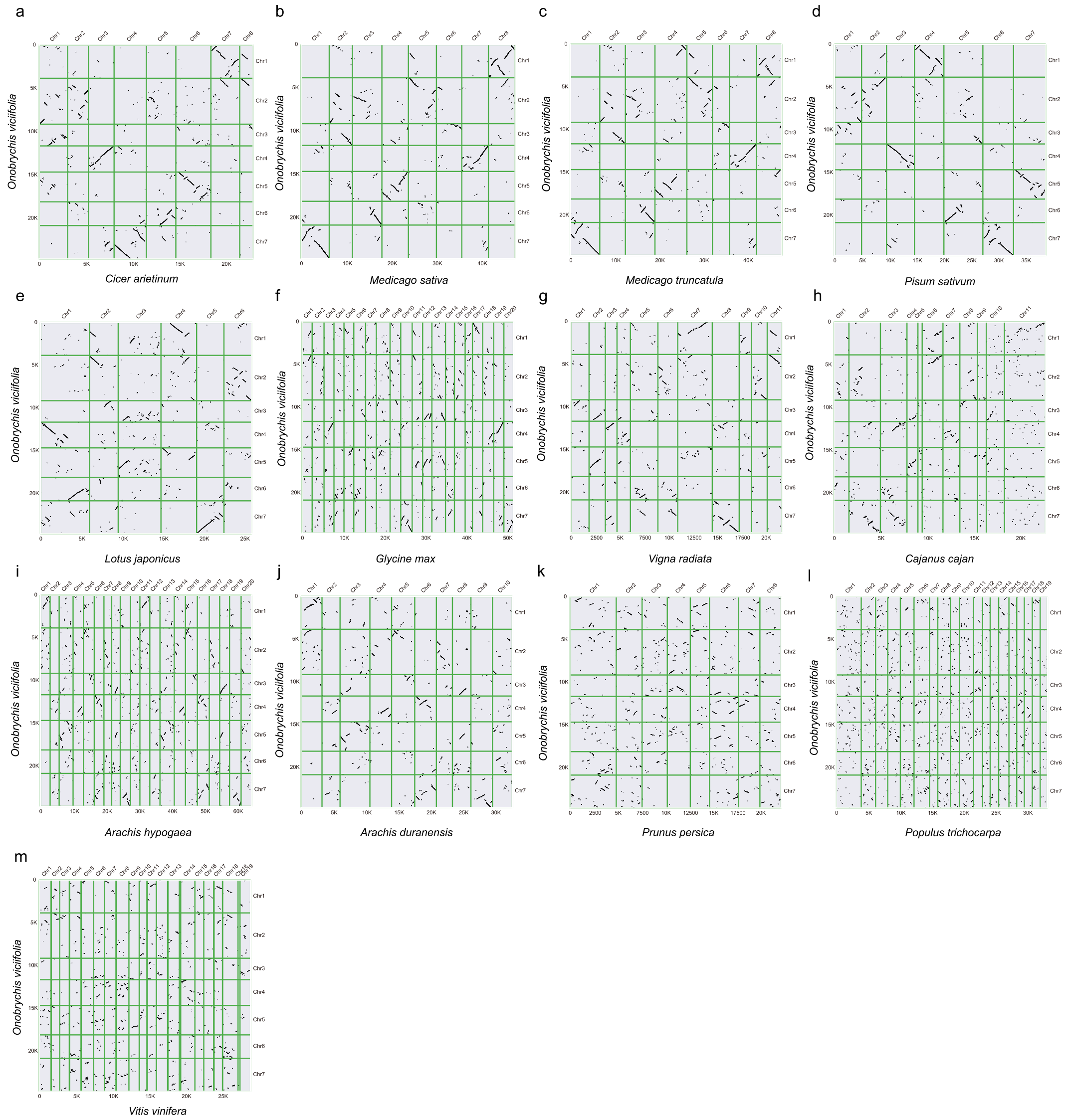

Supplementary Fig. 9 Collinearity of genomes between *O. viciifolia* and selected species. (a) *O. viciifolia* vs *C. arietinum*, (b) *O. viciifolia* vs *M. sativa*, (c) *O. viciifolia* vs *M. truncatula*, (d) *O. viciifolia* vs *P. sativum*, (e) *O. viciifolia* vs *L. japonicus*, (f) *O. viciifolia* vs *G. max*, (g) *O. viciifolia* vs *V. radiata*, (h) *O. viciifolia* vs *C. cajan*, (i) *O. viciifolia* vs *A. hypogaea*, (j) *O. viciifolia* vs *A. duranensis*, (k) *O. viciifolia* vs *P. persica*, (l) *O. viciifolia* vs *P. trichocarpa*, (m) *O. viciifolia* vs *V. vinifera*.

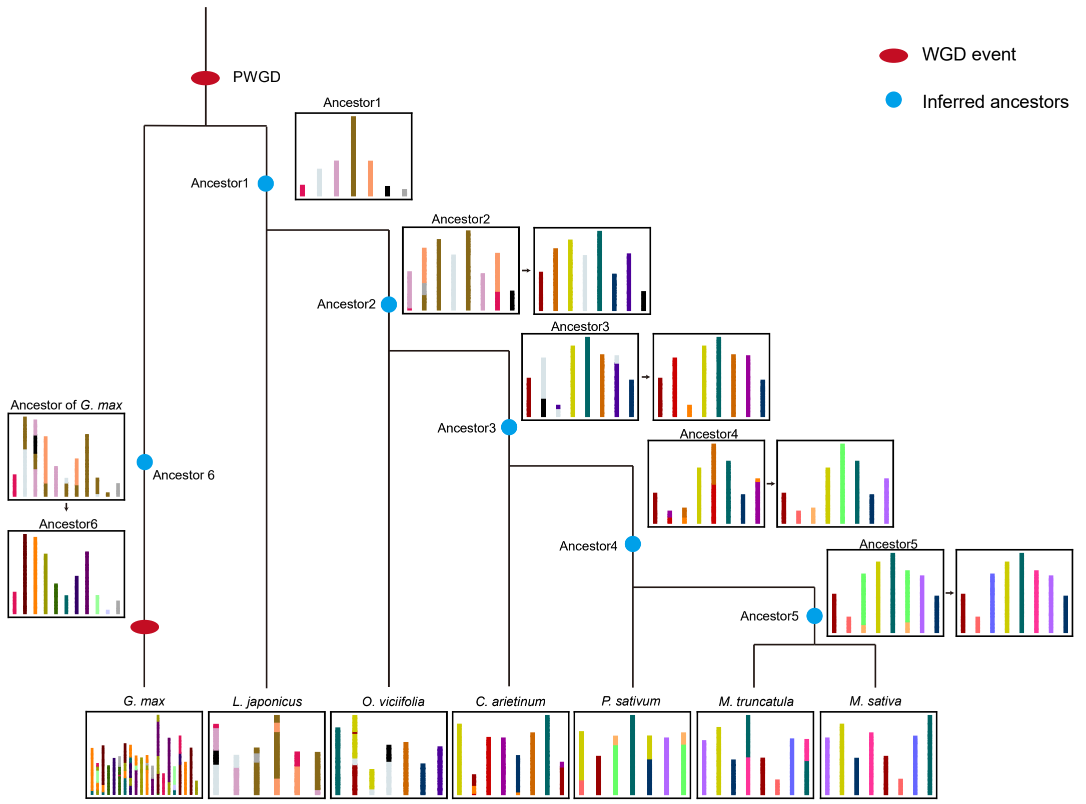

Supplementary Fig. 10 Ancestral karyotype within the selected modern leguminous species. The blue points represented the referred ancestors.

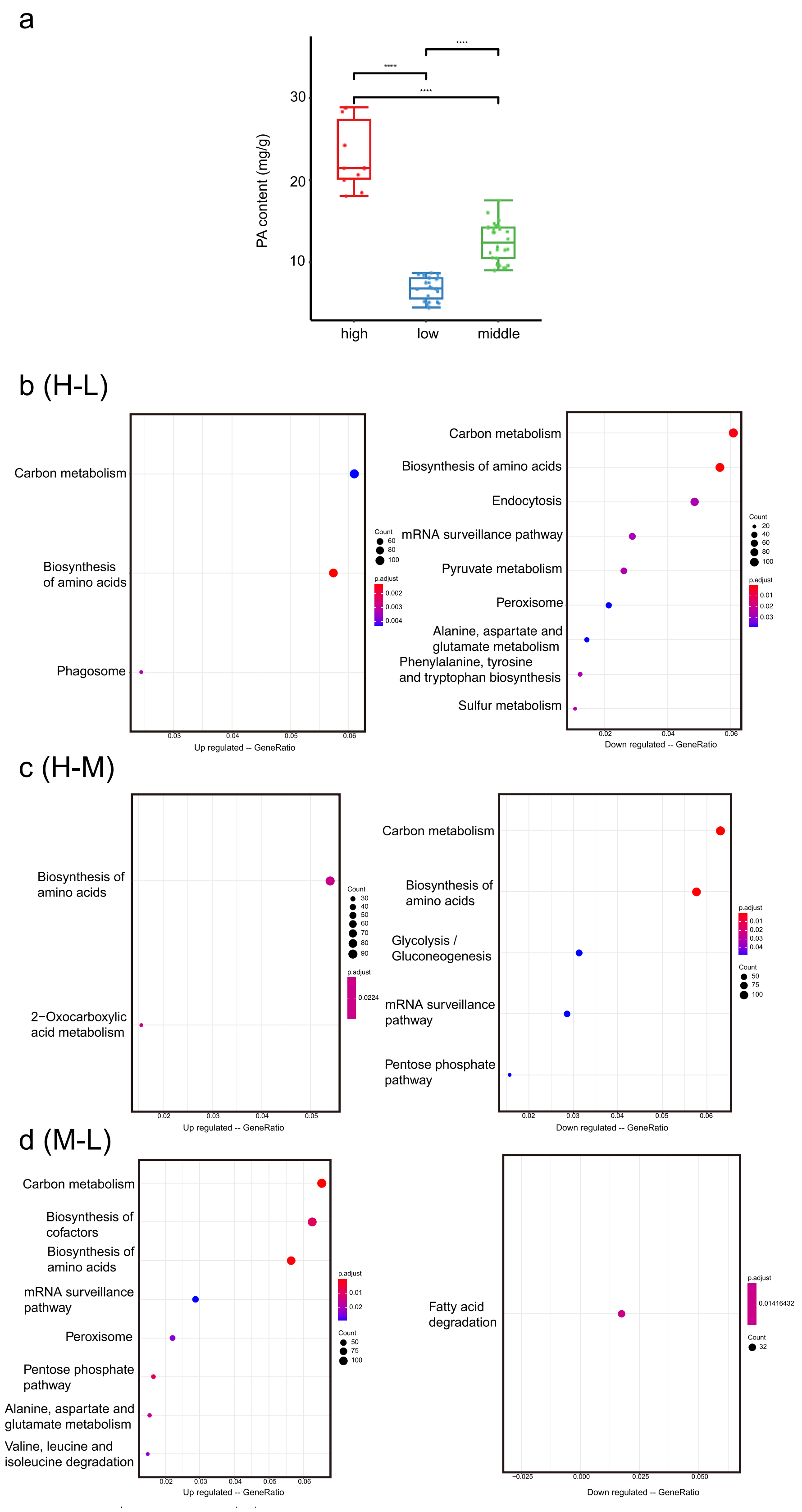

Supplementary Fig. 11 Three groups with different PA contents and KEGG enrichment of DEGs in *O. viciifolia*. (a) The 9 samples were clustered into three groups, based on their high (H), medium (M), and low (L) levels of PA content. Asterisks represent the significance level based on the t test (**** for $P < 0.0001$). (b-d) KEGG enrichment of up- and down-regulated DEGs for the

comparisons of H vs L, H vs M, M vs L.

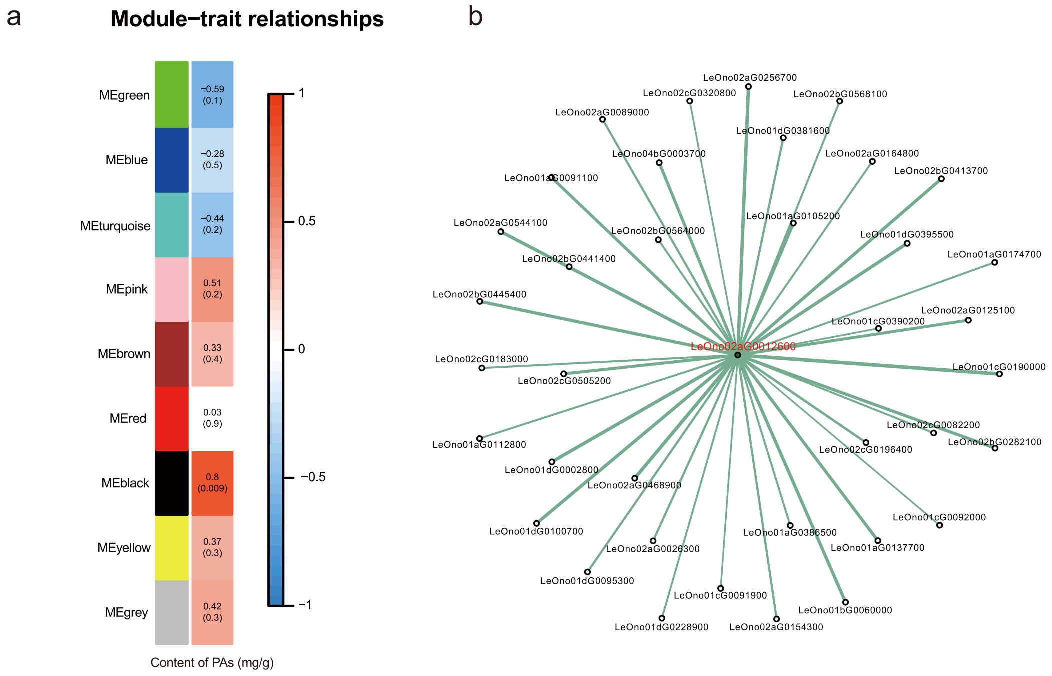

Supplementary Fig. 12 Correlation of gene expression in the three groups with different PA contents by RNA-seq. (a) Correlation between 9 gene modules and 15 phenotypic traits, including PA content. Each cell contains the correlation coefficient and the p-value (in-brackets) between gene modules and the traits. (b) The significant links between the core gene of ANS (LeOno02aG0012600) and the other genes in the gene module MEblack.

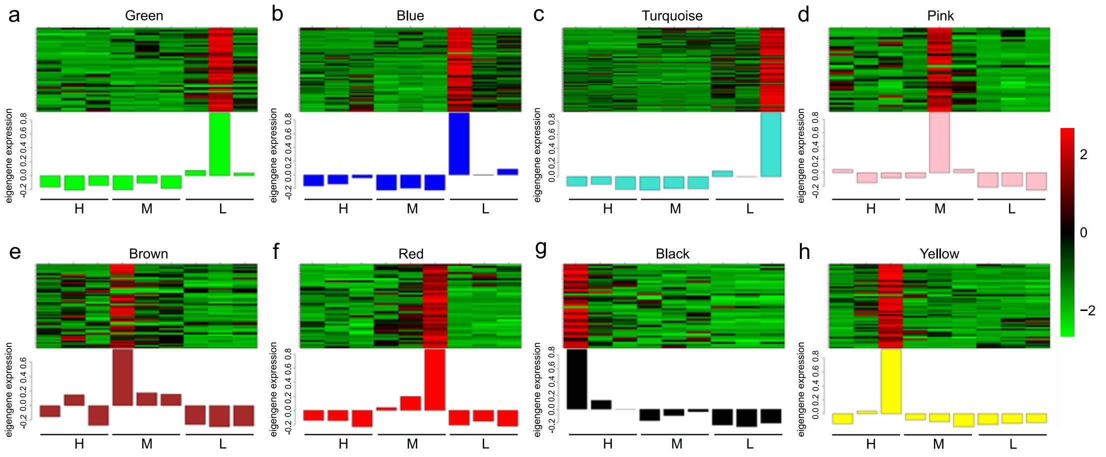

Supplementary Fig. 13 Gene expression levels of different modules in WGCNA. (a) The green represents the gene expression levels in MEgreen modules of WGCNA, (b) blue represents MEblue, (c) turquoise represents MEturquoise, (d) pink presents MEpink, (e) brown presents MEbrown, (f) red presents MEred, (g) black represents MEblack, (h) yellow represents MEyellow. The “H”, “M”, and “L” represent three samples in different PA content levels, respectively.

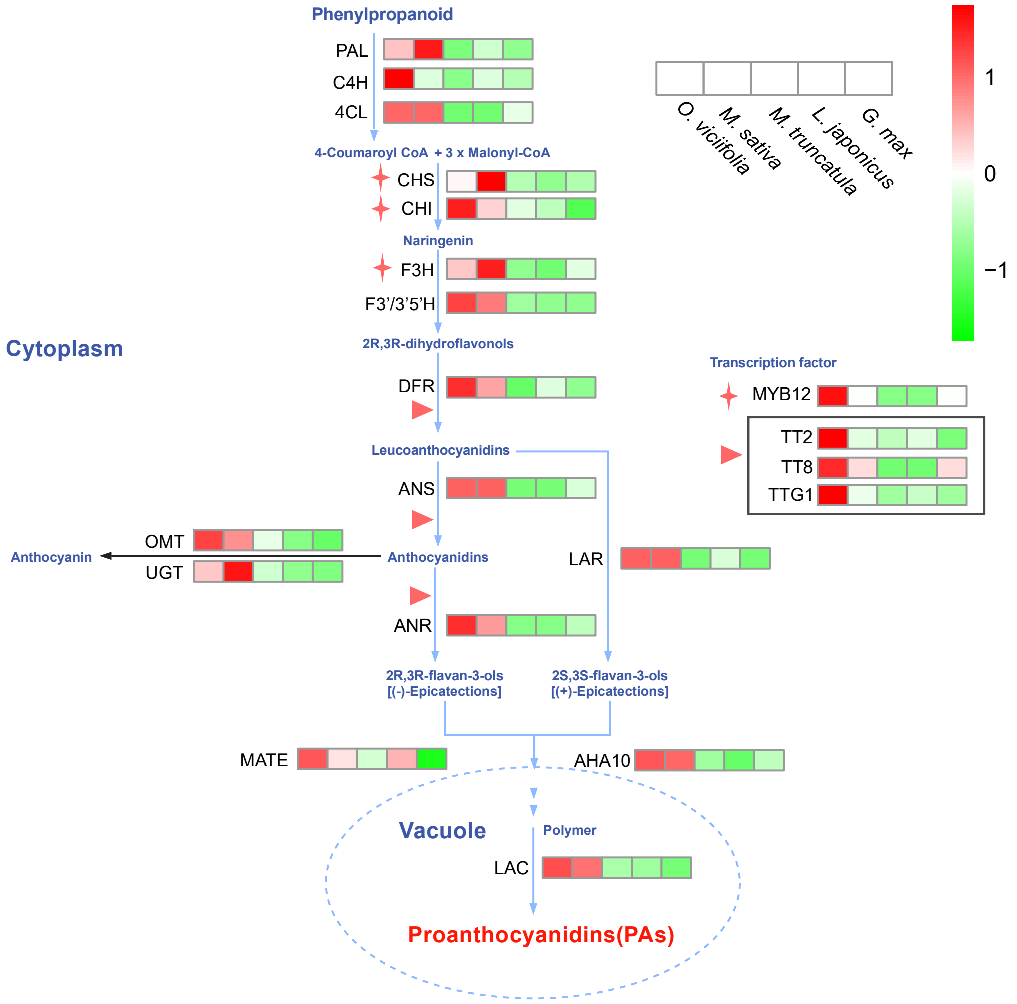

Supplementary Fig. 14 Expansion of genes involved in PA biosynthesis in five Fabales species.

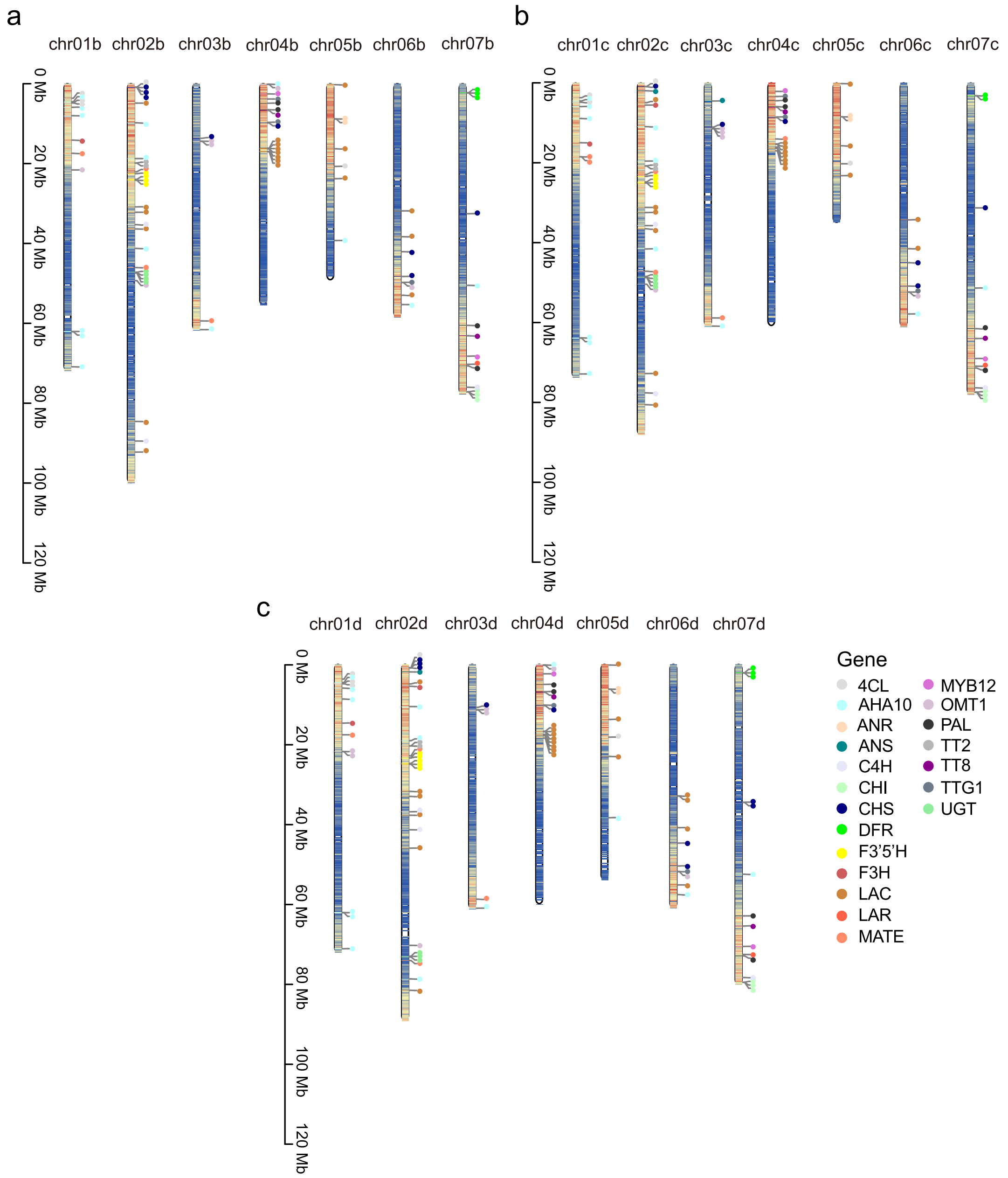

Supplementary Fig. 15 Homologous genes involved in PAs biosynthesis pathway were plotted on the seven chromosomes of haplotype B (a), C (b), and D (c), in *O. viciifolia*.

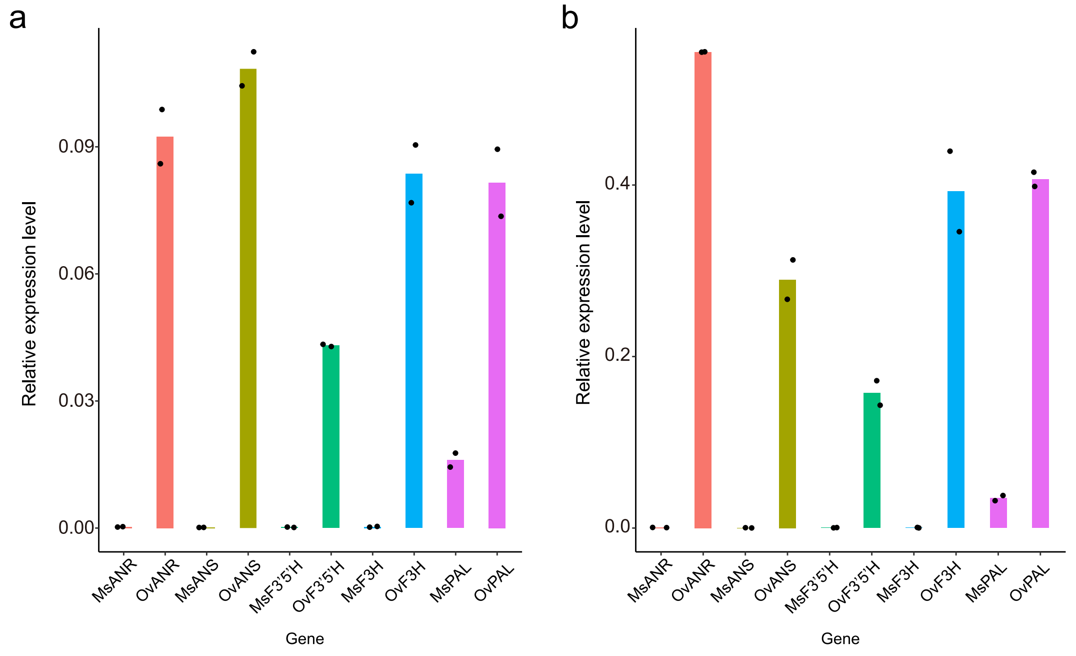

Supplementary Fig. 16 Expressions of selected genes involved in PAs biosynthesis in *O. viciifolia* and *M. sativa* by qRT-PCR (n=2). (a) EF1a as the internal gene, (b) GADPH as the internal gene.

Supplementary Table 1 Sequencing for genome assembly of *Onobrychis viciifolia*

Sequencing platform	Data	Coverage (×)
Oxford Nanopore	200 Gb	~109.89
Illumina NovaSeq	175 Gb	~96.15
Hi-C	300 Gb	~164.84
Total	675 Gb	~370.88

Supplementary Table 2 Comparison of genome assemblies for *M. sativa* cultivar "Zhongmu No.4" and *O. viciifolia*

	***Medicago sativa* cultivar Zhongmu No.4**	***Onobrychis viciifolia* (common sainfoin)**
Estimated genome size by flow cytometry	3068 Mb	1851.75 Mb
Estimated genome size by K-mer	2962 Mb	1821.52 Mb
Total length of assembly	2615.86 Mb	1950.14 Mb
Number of total contigs	49967	1044
Number of unanchored contigs	4864	547
Anchor rate (%)	93.45	99.98
Contig N50	2.06 Mb	10.92 Mb
Max. contig	—	36.37 Mb
GC content of the genome	34.20%	34.62%
Number of annotated genes	146,704	109,998
Repetitive sequences	1558 Mb	1240 Mb
LTR transposable element content	49.28%	36.14%
Complete BUSCOs of assembled genome	1588 (98.40%)	1315 (91.30%)
Complete BUSCOs of annotated proteins	1552 (96.10%)	1400 (97.20%)

Supplementary Table 3 Genome size for *O. viciifolia*

Type	Number	Length	Percent
Pseudo-chromosomes	28	1892.58 Mb	97.03%
Unplaced contigs (non-anchor in pseudo-chromosomes)	547	57.56 Mb	2.95%
Nuclear (pseudo-chromosomes + non-pseudo-chromosomes)	575	1950.14 Mb	99.98%

Supplementary Table 4 Summary for the genome assembly of *O. viciifolia*

**Type**	**Length/Number**
Genome size (nuclear+mt+pt)	1950.60 Mb
Genome size without N	1950.55 Mb
GC content	34.62%
A	637.51 Mb (32.68%)
T	637.66 Mb (32.69%)
C	337.39 Mb (17.30%)
G	337.99 Mb (17.30%)
N	0.04 Mb (0.00%)
Contig number	1044
Max. contig length	36.37 Mb
Mean length	1.87 Mb
N10	31.45 Mb
N50	10.92 Mb
N90	2.19 Mb
Gap number	467
Max. gap length	183 bp
Mean gap length	91 bp

Supplementary Table 5 Summary for the genome assembly of four haplotypes of *O. viciifolia*

		Haplotype A	Haplotype B	Haplotype C	Haplotype D
Size	Total length	482,865,806 bp	475,108,673 bp	458,438,284 bp	478,171,389 bp
	Total length without N	480,853,325 bp	475,098,748 bp	458,428,396 bp	478,161,022 bp
Nucleotide	GC content	34.66%	34.56%	34.60%	34.62%
	A	32.67%	32.72%	32.70%	32.68%
	T	32.67%	32.71%	32.70%	32.70%
	C	17.32%	17.27%	17.29%	17.28%
	G	17.34%	17.29%	17.32%	17.34%
Contig	Contig number	145	111	118	121
	Max.	33,989,258 bp	31,448,785 bp	35,485,197 bp	36,369,481 bp
	Min.	32,382 bp	41,791 bp	44,740 bp	35,706 bp
	Mean	3,316,229 bp	4,280,168 bp	3,884,986 bp	3,951,743 bp
	N10	17,181,229 bp	28,987,351 bp	32,672,205 bp	34,049,222 bp
	N50	9,209,175 bp	13,877,074 bp	12,783,608 bp	13,946,868 bp
	N90	2,850,259 bp	2,988,248 bp	2,804,381 bp	3,040,019 bp
	L10	2	2	2	2
	L50	18	12	11	11
	L90	55	43	38	41
Scaffold	Scaffold number	7	7	7	7
	Max.	88,053,675 bp	100,087,132 bp	88,217,328 bp	89,244,301 bp
	Min.	54,058,121 bp	49,294,000 bp	35,106,131 bp	54,048,558 bp
	Mean	68,695,115 bp	67,872,667 bp	65,491,183 bp	68,310,198 bp
	N10	88,053,675 bp	100,087,132 bp	88,217,328 bp	89,244,301 bp
	N50	78,203,830 bp	71,978,237 bp	73,846,293 bp	72,180,620 bp
	N90	54,058,121 bp	49,294,000 bp	60,930,978 bp	54,048,558 bp
	L10	1	1	1	1
	L50	3	3	3	3
	L90	7	7	6	7
Gap	Gap number	138	104	111	114
	Max.	100 bp	178 bp	183 bp	116 bp
	Min.	1 bp	37 bp	2 bp	10 bp

	Mean	90 bp	95 bp	89 bp	90 bp
Assembly-BUSCO	Complete BUSCOs	1292 (89.7%)	1302 (90.4%)	1298 (90.2%)	1283 (89.1%)
	Complete and single-copy BUSCOs	1207 (83.8%)	1218 (84.6%)	1209 (84.0%)	1202 (83.5%)
	Complete and duplicated BUSCOs	85 (5.9%)	84 (5.8%)	89 (6.2%)	81 (5.6%)
	Fragmented BUSCOs	31 (2.2%)	30 (2.1%)	29 (2.0%)	26 (1.8%)
	Missing BUSCOs	117 (8.1)	108 (7.5%)	113 (7.8%)	131 (9.1%)
	Total BUSCO groups	1440	1440	1440	1440
Annotation-BUSCO	Complete BUSCOs (C)	1315 (91.3%)	1340 (93.0%)	1335 (92.7%)	1318 (91.6%)
	Complete and single-copy BUSCOs (S)	1017 (70.6%)	1050 (72.9%)	1051 (73.0%)	1068 (74.2%)
	Complete and duplicated BUSCOs (D)	298 (20.7%)	290 (20.1%)	284 (19.7%)	250 (17.4%)
	Fragmented BUSCOs (F)	34 (2.4%)	23 (1.6%)	27 (1.9%)	15 (1.0%)
	Missing BUSCOs (M)	91 (6.3%)	77 (5.4%)	78 (5.4%)	107 (7.4%)
	Total BUSCO groups searched	1440	1440	1440	1440
Annotated genomic features	Genes	27299	27438	27238	27162
	Transcript	30388	30002	29568	29439
	Simple_repeat	116953	119111	115011	118366
	LTR/unknown	104441	101402	97537	105224
	LTR/Copia	55357	53807	50677	53506
	LTR/Gypsy	95249	90086	86477	93825
	LINEs	910	874	891	861
	DNAs	295118	292425	286425	299587

Supplementary Table 6 Summary of all repeat elements

Repeat	Superfamily	Number	Length (Mb)	Percentage (%)	Mean_length (bp)
LTR	LTR_total	1,033,972	704.9	36.14	681.74
	LTR_Copia	218,580	150.23	7.7	687.31
	LTR_Gypsy	381,092	339.51	17.41	890.88
	LTR_unknown	434,300	215.16	11.03	495.43
Pararetrovirus	Pararetrovirus	302	0.45	0.02	1479.15
LINE	LINE	3,726	2.05	0.11	550.88
DNA	DNA_total	1,234,695	428.54	21.97	347.09
	DNA_DTA	154,978	52.8	2.71	340.64
	DNA_DTC	138,785	86.38	4.43	622.43
	DNA_DTH	80,651	22.9	1.18	283.93
	DNA_DTM	439,948	141.58	7.26	321.81
	DNA_DTT	46,274	10.4	0.54	224.65
	DNA_Helitron	374,059	114.5	5.87	306.1
Unknown	Unknown	313,300	78.09	4	249.24

Supplementary Table 7 The annotated genes in the genome assembly

Feature	Number	Min.	Max.	Median	Mean
gene	109998	33	373553	2503	3722.5
transcript	130410	33	18864	1383	1564.6
CDS	119930	153	18864	1119	1333.2
exon	719988	3	7950	148	283.4
intron	589578	4	364875	219	539.2

Supplementary Table 8 RNA-seq data downloaded from NCBI for gene modeling

Bioproject	Sample	Accession	Tissue	Development stage	Location
PRJNA612135	SAMN14362542	SRR11310356	leaf	flowering	China
PRJNA612135	SAMN14362541	SRR11310357	leaf	flowering	China
PRJNA612135	SAMN14362540	SRR11310358	leaf	flowering	China
PRJNA612135	SAMN14362539	SRR11310359	leaf	flowering	China
PRJNA612135	SAMN14362538	SRR11310360	leaf	flowering	China
PRJNA612135	SAMN14362537	SRR11310361	leaf	flowering	China
PRJNA315368	SAMN04556712	SRR3229094	shoot and root	Seedling	Switzerland
PRJNA315368	SAMN04556713	SRR3363169	shoot and root	Seedling	United Kingdom
PRJNA315368	SAMN04556714	SRR3363170	shoot and root	Seedling	Czech
PRJNA315368	SAMN04556711	SRR3363171	shoot and root	Seedling	United Kingdom
PRJNA315368	SAMN04556715	SRR3363172	shoot and root	Seedling	Italy
PRJNA436866	SAMN08634426	SRR6804700	14 tissues	Several stages	China

Supplementary Table 9 Mapping rate of all sequenced reads against the genome assembly of *O. viciifolia*

Dataset	Mapped reads	Properly paired reads mapped	Mapped bases	>=1×	>=5×	>=10×	>=20×
Second-generation sequence (short reads)	99.0%	96.9%	99.0%	97.1%	95.9%	94.9%	93.1%
Third-generation sequence (long reads)	97.1%	-	97.5%	99.8%	99.4%	99.1%	98.4%
RNA-Seq reads	91.7%	82.2%	91.7%	24.3%	14.2%	11.2%	8.9%

Supplementary Table 10 BUSCO scoring for the genome assembly and annotated proteins of *O. viciifolia*

	Items	Number
BUSCO evaluation for genome assembly	Complete BUSCOs	1315 (91.3%)
	Complete and single-copy BUSCOs	108 (7.5%)
	Complete and duplicated BUSCOs	1207 (83.8%)
	Fragmented BUSCOs	23 (1.6%)
	Missing BUSCOs	102 (7.1%)
	Total BUSCO groups	1440 (100%)
BUSCO evaluation for annotated proteins	Complete BUSCOs	1400 (97.2%)
	Complete and single-copy BUSCOs	19 (1.3%)
	Complete and duplicated BUSCOs	1381 (95.9%)
	Fragmented BUSCOs	10 (0.7%)
	Missing BUSCOs	30 (2.1%)
	Total BUSCO groups searched	1440 (100%)

Supplementary Table 11 Gene annotations against the databases

	Number	Precentage
**gene**	99518	100%
**Annotated by eggNOG-mapper**		
GO	49897	50.14%
KEGG_KO	47157	47.39%
EC	21021	21.12%
KEGG_Pathway	29300	29.44%
eggNOG	91309	91.75%
COC	97391	97.86%
Unannotated	2127	2.14%
**Annotated by diamond**		
Swiss_Prot	74837	75.20%
TrEMBL	98195	98.67%
NR	98034	98.51%
Arabidopsis thaliana	88412	88.84%
Unannotated	1257	1.30%
**Annotated by InterProScan**		
CDD	34405	34.57%
Pfam	84041	84.45%
SUPERFAMILY	66244	66.56%
PIRSF	7201	7.24%
PANTHER	95594	96.06%
Gene3D	70705	71.05%
Phobius	33808	33.97%
PRINTS	15525	15.60%
TIGRFAM	11062	11.12%
MobiDBLite	38930	39.12%
Coils	16192	16.27%
TMHMM	23902	24.02%
SMART	31745	31.90%
Unannotated	1107	1.11%

Supplementary Table 12 Summary of genomic features in the 28 psuedo-chromosomes

Chr	Genes	LTR	LINEs	DNAs	Simple_repeat
chr1a	4128	42861	153	46279	19320
chr1b	3965	36727	106	46419	18443
chr1c	4194	38451	126	46183	18955
chr1d	4086	37494	148	45664	18935
chr2a	5703	44369	118	53508	22845
chr2b	5886	50885	135	61334	26203
chr2c	5667	42212	164	57472	23418
chr2d	5697	42925	136	61897	23184
chr3a	3040	33916	134	40961	13118
chr3b	3087	32418	166	37457	13356
chr3c	2870	32588	121	38269	12794
chr3d	2877	33026	112	36965	13281
chr4a	3503	32030	108	30572	14121
chr4b	3422	31337	80	30380	13859
chr4c	3609	35117	92	33118	13873
chr4d	3493	34178	62	32544	14625
chr5a	3683	27158	99	38160	14087
chr5b	3736	21737	89	32271	13958
chr5c	3411	12763	74	25351	11718
chr5d	3650	30935	113	33670	13661
chr6a	3024	33828	136	35869	13650
chr6b	3036	31800	110	34919	13583
chr6c	3166	33279	125	36035	13646
chr6d	2975	32719	116	37032	14297
chr7a	4218	40885	162	49769	19812
chr7b	4306	40391	188	49645	19709
chr7c	4321	40281	189	49997	20607
chr7d	4384	41278	174	51815	20383

Supplementary Table 13 The length of genomic features in the 28 psuedo-chromosomes (Mbp)

Chr	Gene	LTR	LINE	DNA	Simple repeat
chr1a	17.280	30.169	0.092	15.667	0.841
chr1b	16.371	25.156	0.058	15.249	0.792
chr1c	16.341	26.355	0.065	15.273	0.819
chr1d	17.387	25.103	0.077	15.399	0.816
chr2a	21.595	29.489	0.062	17.498	0.984
chr2b	22.039	34.001	0.075	20.026	1.154
chr2c	21.404	27.908	0.103	18.641	0.999
chr2d	21.458	28.796	0.072	19.100	0.981
chr3a	10.354	23.510	0.069	16.506	0.605
chr3b	10.284	22.707	0.079	16.606	0.606
chr3c	10.896	23.466	0.066	15.104	0.577
chr3d	10.789	23.751	0.065	15.185	0.607
chr4a	11.871	23.507	0.053	10.878	0.603
chr4b	10.932	22.329	0.049	10.611	0.616
chr4c	10.844	25.827	0.052	12.159	0.591
chr4d	12.008	24.031	0.038	11.891	0.635
chr5a	14.229	17.558	0.052	10.619	0.586
chr5b	13.881	13.907	0.045	10.212	0.584
chr5c	12.875	6.850	0.037	7.182	0.479
chr5d	12.984	19.265	0.069	10.228	0.573
chr6a	11.853	23.990	0.081	12.843	0.585
chr6b	11.576	22.494	0.064	12.850	0.576
chr6c	11.241	24.417	0.068	12.958	0.596
chr6d	11.439	23.960	0.067	13.177	0.630
chr7a	16.257	27.912	0.104	16.279	0.851
chr7b	16.373	27.243	0.104	16.474	0.847
chr7c	16.734	26.645	0.094	16.273	0.899
chr7d	16.500	28.277	0.101	16.796	0.896

Supplementary Table 14 Annotation information of four haplotypes

	Haploid A	Haploid B	Haploid C	Haploid D
Number of genes	27299	27438	27238	27162
Number of annotated genes	24837	24841	24742	24725
COG	97.92%	97.81%	97.91%	97.87%
GO	50.18%	50.20%	50.11%	50.15%
EC	21.09%	20.89%	21.21%	21.27%
KEGG_KO	41.07%	41.22%	41.09%	41.45%
KEGG_Pathway	23.27%	23.43%	23.24%	23.77%
eggNOG	91.77%	91.86%	91.78%	91.66%
Swiss_Prot	75.42%	75.25%	75.09%	75.21%
TrEMBL	98.69%	98.60%	98.71%	98.72%
NR	98.53%	98.45%	98.53%	98.57%
A.thaliana	89.09%	88.80%	88.90%	88.78%
CDD	34.42%	34.58%	35.04%	34.51%
Coils	16.46%	16.24%	16.31%	16.17%
Gene3D	70.87%	71.10%	71.17%	71.27%
MobiDBLite	39.18%	39.10%	39.44%	39.01%
PANTHER	96.15%	96.12%	96.20%	95.90%
Pfam	84.50%	84.62%	84.49%	84.31%
Phobius	34.13%	33.71%	34.23%	33.88%
PIRSF	7.28%	7.26%	7.27%	7.14%
PRINTS	15.55%	15.58%	15.44%	15.85%
SMART	31.78%	31.96%	32.20%	31.85%
SUPERFAMILY	66.35%	66.52%	66.75%	66.82%
TIGRFAM	11.02%	11.18%	11.02%	11.18%
TMHMM	24.24%	23.82%	24.17%	23.86%

Supplementary Table 15 KEGG analysis for the genes specifically found in haploids B, C, and D, compared to haploid A

**Haploid**	**Description**	**GeneRatio**	**p.adjust**
Haploid B	Oxidative phosphorylation	28/626	1.44E-06
	Fructose and mannose metabolism	16/626	0.001562475
Haploid C	Oxidative phosphorylation	23/581	3.73E-05
	Diterpenoid biosynthesis	14/581	0.000229412
	Flavone and flavonol biosynthesis	5/581	0.012283588
	Arginine and proline metabolism	12/581	0.017199877
Haploid D	Fructose and mannose metabolism	24/640	2.41E-06
	Oxidative phosphorylation	27/640	1.24E-05

Supplementary Table 16 LAI of five selected legume species

**Species (Version)**	**raw LAI**	**LAI**
C. arietinum (ASM33114v1)	2.77	3.67
C. cajan (C.cajan_V1.1)	2.85	3.79
M. truncatula (MtrunA17r5.0-ANR)	10.70	10.72
V. radiata (Vradiata_ver6)	2.35	6.50
O. viciifolia Haplotype A	10.90	6.49
O. viciifolia Haplotype B	10.90	5.79
O. viciifolia Haplotype C	11.28	7.05
O. viciifolia Haplotype D	10.11	4.48

Supplementary Table 17 Quality value estimation for the whole genome

	Quality value	Error rate
Whole genome	33.3884	0.000458311
Haplotype A	33.527	0.00044392
Haplotype B	33.879	0.000409352
Haplotype C	33.6277	0.000433741
Haplotype D	33.7472	0.000421964

Note: quality values were calculated by using Merqury based on short reads.

Supplementary Table 18 KEGG enrichment analysis for the unique gene families in *O. viciifolia*

Description	GeneRatio	pvalue	p.adjust	qvalue
Zeatin biosynthesis	18/181	8.54645E-16	6.40984E-14	5.03791E-14
Taurine and hypotaurine metabolism	5/181	1.18265E-05	0.000443495	0.000348572
Alanine, aspartate and glutamate metabolism	8/181	2.50981E-05	0.000627452	0.000493156
Mismatch repair	8/181	4.23236E-05	0.000793568	0.000623716
Butanoate metabolism	5/181	6.62994E-05	0.000994491	0.000781635
DNA replication	8/181	0.000137345	0.001716807	0.00134935
Pentose and glucuronate interconversions	11/181	0.000409714	0.004389796	0.003450225
beta-Alanine metabolism	5/181	0.002203289	0.020655832	0.016234759
Nucleotide excision repair	6/181	0.005511371	0.04592809	0.036097867

Supplementary Table 19 KEGG enrichment analysis for the retained genes of *O. viciifolia* experienced PWGD event

Description	GeneRatio	pvalue	p.adjust	qvalue
Plant hormone signal transduction	76/1285	7.28919E-08	8.89282E-06	8.1332E-06
Endocytosis	45/1285	0.000145341	0.007423673	0.006789554
MAPK signaling pathway - plant	41/1285	0.000182549	0.007423673	0.006789554

Supplementary Table 20 GO analysis in biological process for the retained genes of *O. viciifolia* experienced PWGD event

Description	GeneRatio	p.adjust
Intracellular signal transduction	93/1783	6.01146E-05
Response to gibberellin	43/1783	0.000235707
Response to ethylene	57/1783	0.000993085
Molecular transducer activity	67/1783	0.000993085
Obsolete transcription regulator recruiting activity	32/1783	0.000993085
Obsolete RNA polymerase II transcription regulator Recruiting activity	32/1783	0.000993085
DNA-binding transcription factor activity, RNA Polymerase II-specific	46/1783	0.001197222
Response to abscisic acid	107/1783	0.001254353
Hormone-mediated signaling pathway	99/1783	0.001494376
Obsolete transcription factor activity, RNA Polymerase II transcription factor binding	33/1783	0.001494376
Signaling receptor activity	61/1783	0.001494376
Cellular response to acid chemical	81/1783	0.001494376
Obsolete transcription factor activity, transcription Factor binding	34/1783	0.001494376
Response to water deprivation	83/1783	0.001549386
Response to water	83/1783	0.00201093
Response to chitin	41/1783	0.002456404
Response to organonitrogen compound	62/1783	0.002654855
Transmembrane receptor protein serine/threonine Kinase activity	46/1783	0.003594933
Enzyme linked receptor protein signaling pathway	47/1783	0.003783957
Transmembrane receptor protein serine/threonine Kinase signaling pathway	46/1783	0.003783957
Response to cold	77/1783	0.00485397
Regulatory region nucleic acid binding	76/1783	0.005471589
Reproductive shoot system development	80/1783	0.005540254
Obsolete transcription factor activity, protein binding	34/1783	0.006164479
Flower development	77/1783	0.006164479
Transmembrane signaling receptor activity	52/1783	0.008616226
Transmembrane receptor protein kinase activity	47/1783	0.01015707
Root system development	101/1783	0.010385832
Abscisic acid-activated signaling pathway	32/1783	0.014325512
Root development	100/1783	0.014325512
Cellular response to ethylene stimulus	24/1783	0.015166633
Root epidermal cell differentiation	31/1783	0.026505843

Response to auxin	64/1783	0.029127263
Regulation of reproductive process	65/1783	0.029211457
Peptidyl-serine phosphorylation	20/1783	0.029469616
Peptidyl-serine modification	20/1783	0.036149313
Plant organ morphogenesis	83/1783	0.038145184
Phosphorelay signal transduction system	22/1783	0.048255603

Supplementary Table 21 Statistics of chromosomal fissions and fusions in the processes of karyotype evolution

The species evolved from Ancestor1	Fission events	Fusion events
L. japonicus	29	30
O. viciifolia	16	16
C. arietinum	24	23
P. sativum	26	26
M. truncatula	21	20
M. sativa	22	21

Supplementary Table 22 The 41 sainfoin accessions for phenotypic traits

No.	Accession	Region
1	PI 306693 88i SD	Emilia-Romagna Italy
2	PI 502554 87i SD	Russian Federation
3	PI 319062 88i SD	Spain
4	PI 318604 88i SD	Switzerland
5	PI 234822 88i SD	Switzerland
6	PI 236486 2017i SD	Turkey
7	PI 225728 2017i SD	Turkey
8	PI 206459 88i SD	Turkey
9	PI 206458 88i SD	Turkey
10	PI 205202 88i SD	Turkey
11	PI 205200 88i SD	Turkey
12	PI 204594 88i SD	Turkey
13	PI 200872 90i SD	Turkey
14	PI 273785 88i SD	Ukraine
15	PI 639688 20110 SD	Wyoming United states
16	PI 201512 2007i SD	—
17	PI 192995 89i SD	—
18	PI 316296 87i SD	Former Soviet Union
19	PI 313060 87i SD	Former Soviet Union
20	PI 313054 87i SD	Former Soviet Union
21	PI 313053 87i SD	Former Soviet Union
22	PI 258777 88i SD	Former Soviet Union
23	PI 258774 88i SD	Former Soviet Union
24	PI 258771 88i SD	Former Soviet Union
25	PI 258768 88i SD	Former Soviet Union
26	PI 258767 88i SD	Former Soviet Union
27	PI 110404 88i SD	Former Soviet Union
28	PI 110400 88i SD	Former Soviet Union
29	PI 239959 88i SD	Iran
30	PI 2399598 2011i SD	Iran
31	PI 229613 82i SD	Iran
32	PI 229613 88i SD	Iran
33	PI 227373 82i SD	Iran
34	PI 223389 88i SD	Iran
35	PI 201865 2007i SD	Iran
36	PI 440577 86i SD	Kazakhstan
37	PI 251160 2010i SD	Macedonia
38	PI 338651 88i SD	Morocco
39	PI 494668 86i SD	Romania

40	PI 494667 86i SD	Romania
41	PI 319713 88i SD	Romania

Supplementary Table 23 Phenotype details of 59 individuals for 41 sainfoin accessions

PI	CPA	RNA-seq	PI	CPA	RNA-seq
PI 110400 88i SD	28.88	S1	PI 225728 2017i SD	9.61	-
PI 338651 88i SD	28.83	S2	PI 234822 88i SD	9.35	-
PI 494667 86i SD	28.37	S3	PI 201865 2007i SD	9.35	-
PI 205202 88i SD	24.61	-	PI 319713 88i SD	9.03	-
PI 205200 88i SD	21.79	-	PI 229613 88i SD	8.71	-
PI 258767 88i SD	21.49	-	PI 239959 88i SD	8.56	-
PI 251160 2010i SD	20.67	-	PI 258768 88i SD	8.55	-
PI 110400 88i SD	20.03	-	PI 306693 88i SD	8.5	-
PI 258771 88i SD	18.52	-	PI 204594 88i SD	8.49	-
PI 110404 88i SD	18.08	-	PI 494668 86i SD	8.15	-
PI 200872 90i SD	18.01	-	PI 204594 88i SD	8.01	-
PI 319062 88i SD	16.07	-	PI 338651 88i SD	7.97	-
PI 206459 88i SD	15.13	-	PI 223389 88i SD	7.57	-
PI 319062 88i SD	14.73	-	PI 236486 2017i SD	7.53	-
PI 2399598 2011i SD	14.4	S4	PI 110404 88i SD	6.99	-
PI 258767 88i SD	14.3	S5	PI 440577 86i SD	6.86	-
PI 2399598 2011i SD	14.07	S6	PI 313060 87i SD	6.82	-
PI 227373 82i SD	14.03	-	PI 258774 88i SD	6.79	-
PI 223389 88i SD	13.74	-	PI 313053 87i SD	6.76	-
PI 236486 2017i SD	13.65	-	PI 273785 88i SD	6.67	-
PI 494667 86i SD	13.65	-	PI 200872 90i SD	6.51	-
PI 110404 88i SD	12.87	-	PI 639688 20110 SD	6.43	-
PI 258771 88i SD	11.94	-	PI 502554 87i SD	5.95	-
PI 236486 2017i SD	11.61	-	PI 258777 88i SD	5.25	-
PI 639688 20110 SD	11.57	-	PI 229613 82i SD	5.22	-
PI 313054 87i SD	11.53	-	PI 318604 88i SD	5.14	-
PI 201512 2007i SD	11.18	-	PI 316296 87i SD	5.04	S7
PI 110404 88i SD	10.58	-	PI 502554 87i SD	4.9	S8
PI 110404 88i SD	9.76	-	PI 192995 89i SD	4.5	S9
PI 205200 88i SD	9.65	-	–	–	–

Note: Nine lines were selected to do RNA-Seq in leaves, which are highlighted in red. CPA:Content of PAs (mg/g); DMW: Dry matter weight (g); NH: Natural height (cm); VH: Vertical height (cm); BN: Branch number; CL: Compound leaf number; LN: Leaflet number; SH: Shoot height (cm); SD: Stem diameter (mm); CLL: Compound leaf length (cm); LA: LeafLet area (cm2); LP: Leaflet perimeter (cm); LL: Leaflet length (cm); LW: Leaflet width (cm); LR: Leaflet roundness.

Supplementary Table 24 RNA-seq data and their alignment rates

Alignment Rate	S1	S2	S3	S4	S5	S6	S7	S8	S9
Raw pair-end reads in RNA-Seq	19,223,311	26,346,213	21,137,035	22,948,386	26,122,305	22,763,170	28,294,622	23,141,563	22,842,530
Overall alignment rate against the whole genome assembly	90.88%	90.96%	90.33%	92.66%	91.89%	93.30%	90.13%	90.52%	91.08%
Alignment rate against haploid a	82.68%	80.86%	82.58%	82.36%	82.76%	83.49%	81.17%	81.69%	82.71%
Alignment rate against haploid b	85.65%	85.31%	86.16%	83.06%	83.81%	83.36%	85.04%	84.56%	85.93%
Alignment rate against haploid c	83.81%	82.83%	83.82%	82.10%	82.74%	82.12%	82.08%	82.69%	83.38%
Alignment rate against haploid d	85.08%	85.19%	86.22%	82.72%	83.20%	82.73%	85.19%	84.48%	86.11%

Supplementary Table 25 The DEGs in the MEblack gene module showing a significant correlation with high PA content

Gene	Gene ID annotated in *Arabidopsis thanliana* and *Medicago truncatula*	Function	Name
LeOno01bG0060000	NP_850047.1	Ribosomal protein L35	plastid ribosomal protein L35
LeOno02cG0320800	NP_191670.1	ribosomal protein S27	ARS27A
LeOno02bG0413700	NP_193086.1	transmembrane protein	T6G15.50
LeOno01dG0100700	NP_201090.1	Phosphoglycerate mutase family protein	MQB2.16
LeOno02aG0164800	NP_001185189.1	transmembrane protein	AT1G50732
LeOno01dG0095300	NP_201078.1	Integral membrane HPP family protein	MQB2.4
LeOno02bG0441400	NP_186902.1	TCP-1/cpn60 chaperonin family protein	F16B3.16
LeOno02bG0282100	NP_194433.1	fatty acid desaturase A	F10M23.370
LeOno01dG0228900	NP_001117487.1	linoleate 9S-lipoxygenase-4 protein, putative (DUF581)	AT1G53903
LeOno02aG0026300	NP_001318886.1	nitrogen fixation S (NIFS)-like 1	ATNFS1
LeOno04bG0003700	NP_567198.1	ATPase, F1 complex, OSCP/delta subunit protein	AT4G00895
LeOno02cG0196400	NP_001331391.1	NagB/RpiA/CoA transferase-like superfamily protein	MFC16.20
LeOno02cG0082200	NP_001030831.1	breast basic conserved 1	40S RIBOSOMAL PROTEIN
LeOno01cG0390200	NP_001327480.1	RING/U-box superfamily protein	AT3G25030
LeOno01aG0386500	NP_177245.1	MLP-like protein 43	F15H11.12
LeOno02aG0468900	NP_001319452.1	Ribosomal protein S7e family protein	F16B3.19
LeOno01dG0381600	NP_177241.3	MLP-like protein 31	F15H11.9
LeOno02aG0154300	NP_190560.1	Translation protein SH3-like family protein	F3A4.4
LeOno02aG0012600	NP_001031700.1	leucoanthocyanidin dioxygenase	ANS
LeOno02bG0564000	NP_190560.1	Translation protein SH3-like family protein	F3A4.4
LeOno02cG0505200	NP_177217.1	Ribosomal protein L18e/L15 superfamily protein	F24J13.17
LeOno02bG0445400	NP_198590.1	Calcium-dependent lipid-binding (CaLB domain) family protein	K12B20.22
LeOno01cG0190000	XP_013445079.1	repetitive proline-rich cell wall protein 1-like	LOC25501074
LeOno02bG0568100	NP_001331062.1	Ribonuclease E inhibitor RraA/Dimethylmenaquinone methyltransferase	K24C1.7
LeOno01dG0002800	NP_199787.2	Leucine-rich repeat protein kinase family protein	K2I5.13
LeOno02aG0256700	NP_177605.2	Gibberellin-regulated family protein	F1M20.35
LeOno02aG0544100	NP_190560.1	Translation protein SH3-like family protein	F3A4.4
LeOno01dG0395500	NP_001326155.1	Ribosomal L18p/L5e family protein	AT3G45020
LeOno01aG0137700	NP_191346.1	sumo conjugation enzyme 1	AHUS5
LeOno01aG0112800	NP_001077760.1	Class I peptide chain release factor	AT1G62850
LeOno02aG0089000	XP_039684440.1	uncharacterized LOC120577328	LOC120577328
LeOno01aG0174700	NP_180462.2	Leucine-rich repeat protein kinase family protein	T9I4.4
LeOno02cG0183000	NP_196772.1	elongation factor 1-beta 1	MXC9.7
LeOno01aG0105200	NP_196945.1	hypothetical protein	F18O22.200
LeOno02aG0125100	-	-	-

LeOno01cG0092000	-	ncRNA	-
LeOno01cG0091900	-	ncRNA	-
LeOno01aG0091100	-	ncRNA	-

Supplementary Table 26 The reference genes used for gene copy number determination in autotetraploid *O. viciifolia* and *M. sativa*

Gene	Gene ID	Species
AtPAL	AT2G37040	*Arabidopsis thaliana*
AtC4H	AT2G30490	*Arabidopsis thaliana*
At4Cl5	AT3G21230.1	*Arabidopsis thaliana*
AtCHI	AT3G55120.1	*Arabidopsis thaliana*
AtCHS	AT5G13930.1	*Arabidopsis thaliana*
F3H	AT3G51240.1	*Arabidopsis thaliana*
F3'5'H	NC_012012.3	*Vitis vinifera*
DFR	AT5G42800	*Arabidopsis thaliana*
AtANS	AT4G22880	*Arabidopsis thaliana*
ANR	VIT_00006396001	*Vitis vinifera*
UGT	AT4G15280	*Arabidopsis thaliana*
OMT1	AT5G54160	*Arabidopsis thaliana*
LAR1	VIT_00011958001	*Vitis vinifera*
MATE	NC_027769.2	*Glycine max*
AHA10	AT1G17260	*Arabidopsis thaliana*
ATLAC15	AT5G48100	*Arabidopsis thaliana*
TT2	AT5G35550	*Arabidopsis thaliana*
TT8	AT4G09820	*Arabidopsis thaliana*
TTG1	AT5G24520	*Arabidopsis thaliana*
MYB12	AT2G47460	*Arabidopsis thaliana*

Supplementary Table 27 Relative expressions of genes involved in PA biosynthesis pathway in leaves of *O. viciifolia* and *M. sativa*.

	GADPH				CYP				EF1a			
	H-Sainfoin	M-Sainfoin	L-Sainfoin	Alfalfa	H-Sainfoin	M-Sainfoin	L-Sainfoin	Alfalfa	H-Sainfoin	M-Sainfoin	L-Sainfoin	Alfalfa
PAL	1.0733	0.9267	0.8458	0.1604	0.1308	0.1146	0.0947	1.5503	0.1698	0.1686	0.1152	0.9774
C4H	0.6272	0.5764	0.7121	0.0257	0.0764	0.0712	0.0797	0.2480	0.0992	0.1049	0.0970	0.1563
4Cl	0.2112	0.1731	0.2571	0.0319	0.0257	0.0214	0.0288	0.3083	0.0334	0.0315	0.0350	0.1944
CHI	0.5970	0.5092	0.5810	0.0005	0.0727	0.0629	0.0651	0.0044	0.0944	0.0926	0.0791	0.0028
CHS	4.1487	3.0978	3.5476	0.0002	0.5055	0.3829	0.3972	0.0020	0.6563	0.5635	0.4832	0.0013
F3H	0.6272	0.3646	0.3959	0.0009	0.0764	0.0451	0.0443	0.0089	0.0992	0.0663	0.0539	0.0056
F3'5'H	0.5948	0.2037	0.3445	0.0007	0.0725	0.0252	0.0386	0.0069	0.0941	0.0371	0.0469	0.0044
DFR	0.1616	0.0407	0.0925	0.0000	0.0197	0.0050	0.0104	0.0000	0.0256	0.0074	0.0126	0.0000
ANS	1.0560	0.2363	0.7841	0.0001	0.1287	0.0292	0.0878	0.0008	0.1671	0.0430	0.1068	0.0005
ANR	1.2651	0.2994	0.5887	0.0000	0.1541	0.0370	0.0659	0.0000	0.2001	0.0545	0.0802	0.0000
UGT	1.3254	1.9878	1.9357	0.0056	0.1615	0.2457	0.2168	0.0541	0.2097	0.3616	0.2637	0.0341
OMT1	1.5733	0.7026	2.3445	0.0212	0.1917	0.0869	0.2625	0.2046	0.2489	0.1278	0.3193	0.1290
LAR	0.0409	0.0428	0.0308	0.0000	0.0050	0.0053	0.0035	0.0000	0.0065	0.0078	0.0042	0.0000
MATE	0.1164	0.1283	0.1080	0.0305	0.0142	0.0159	0.0121	0.2943	0.0184	0.0233	0.0147	0.1855
AHA10	0.9935	1.1202	1.2288	0.0855	0.1211	0.1385	0.1376	0.8266	0.1572	0.2038	0.1674	0.5211
LAC	0.1552	0.1283	0.1722	0.0009	0.0189	0.0159	0.0193	0.0091	0.0245	0.0233	0.0235	0.0057
TT2	0.0625	0.0163	0.0283	0.0000	0.0076	0.0020	0.0032	0.0000	0.0099	0.0030	0.0039	0.0000
TT8	0.0129	0.0143	0.0103	0.0007	0.0016	0.0018	0.0012	0.0071	0.0020	0.0026	0.0014	0.0045
TTG1	0.2069	0.1548	0.2211	0.0061	0.0252	0.0191	0.0248	0.0594	0.0327	0.0282	0.0301	0.0375
MYB	0.1013	0.0876	0.0540	0.0001	0.0123	0.0108	0.0060	0.0012	0.0160	0.0159	0.0074	0.0007

Note: three internal genes of GADPH, CYP, and EF1a were selected to do the comparison of relative expression of genes involved in PA biosynthesis pathway, in three groups of sainfoin with high (H-Sainfoin), medium (M-Sainfoin), and low (L-Sainfoin) PA contents, and alfalfa cultivar "Zhongmu No.1". RNA-seq data for alfalfa leaves was downloaded from NCBI, with accession number of PRJNA795295.

Supplementary Table 28 Primers for gene expressions in PA biosynthesis pathway by using qRT-PCR

Name	Primer sequences	Note
OvGADPH-F	CACAACCAACTGCCTTGCTC	Internal gene GADPH in *O. viciifolia*
OvGADPH-R	CTGGGTGGCAGTGATGGAAT	Internal gene GADPH in *O. viciifolia*
MsGADPH-F	CATTCCGTGTCCCAACTGTG	Internal gene GADPH in *M. sativa*
MsGADPH-R	TTCAAGTTGCCCTCGGACTC	Internal gene GADPH in *M. sativa*
OvEF1a-F	AAGCGTGGTTTCGTTGCATC	Internal gene EF1a in *O. viciifolia*
OvEF1a-R	TGTGGGATGTGTGGCAATCA	Internal gene EF1a in *O. viciifolia*
MsEF1a-F	AAGCGTGGTTTCGTTGCATC	Internal gene EF1a in *M. sativa*
MsEF1a-R	GTGAGAGGTGTGGCAATCGA	Internal gene EF1a in *M. sativa*
OvPAL-F	GCTGTTGGTTCTGGTTTGGC	Gene PAL in *O. viciifolia*
OvPAL-R	ACCTTGCATCACTTCAGCGA	Gene PAL in *O. viciifolia*
MsPAL-F	CGGGTGAGGAGTGTGACAAA	Gene PAL in *M. sativa*
MsPAL-R	CCGTTCCACTCTCCCAAACA	Gene PAL in *M. sativa*
OvANR-F	CGCGCACATATATTCCTGGC	Gene ANR in *O. viciifolia*
OvANR-R	ACTGAGGGTATCGCTTGCTG	Gene ANR in *O. viciifolia*
MsANR-F	TGTGGCAGAGAAAGAATCAGCT	Gene ANR in *M. sativa*
MsANR-R	ACTGAGGGTATCGTTTGCTGA	Gene ANR in *M. sativa*
OvF3H-F	GCCAATTGAGGGTGCTTTCG	Gene F3H in *O. viciifolia*
OvF3H-R	CTGGGTTCTGGAATGTGGCT	Gene F3H in *O. viciifolia*
OvF35H-F	ACAACGACGCCTCCAAGAAT	Gene F3'5'H in *O. viciifolia*
OvF35H-R	CGAGGCAAGTTTAGTGGGGT	Gene F3'5'H in *O. viciifolia*
OvANS-F	GGAGTTGAAGCCCACACAGA	Gene ANS in *O. viciifolia*
OvANS-R	CCGGGGACACATTTAGCAGT	Gene ANS in *O. viciifolia*
MsF3H-F	CAGGGAGAAGCTGTGAAGGA	Gene F3H in *M. sativa*
MsF3H-R	ATCCTTCTGGCTTGTCTGGC	Gene F3H in *M. sativa*
MsF35H-F	GGGCTTCGTATGGTCCAACT	Gene F3'5'H in *M. sativa*
MsF35H-R	ACTGATAATGGCACGGCTCG	Gene F3'5'H in M. sativa
MsANS-F	GCTGGTGGAATGGAAGAGCT	Gene ANS in *M. sativa*
MsANS-R	GGGCTTCAACTCCAAGTGCT	Gene ANS in *M. sativa*
