# Supplementary material for: A chromosome-level genome assembly for Onobrychis viciifolia reveals gene copy number gain underlying enhanced proanthocyanidin biosynthesis
Source: Commun Biol. 2024 Jan 5;7:19. doi: 10.1038/s42003-023-05754-6 (PMC10770414; doi:10.1038/s42003-023-05754-6)
Supplement: Supplementary file 2 — Description of Additional Supplementary Files [file 42003_2023_5754_MOESM2_ESM.pdf]

Description of Additional Supplementary Files

File name: Supplementary Data 1
Description: The source data of fig. 1d that GC content vs. chromosome number of Fabaceae and non-Fabaceae species.

File name: Supplementary Data 2
Description: The source data of fig. 2c that LTR insertion time and density in six Fabales plants.

File name: Supplementary Data 3
Description: The source data of fig. 3a that Distribution of synonymous substitution rates (Ks) of homologous gene pairs between O. viciifolia and other species.

File name: Supplementary Data 4
Description: The source data of fig. 3b that Syntenic depths between O. viciifolia and C. arietinum.
